# Coding palindromes in mitochondrial genes of Nematomorpha

**DOI:** 10.1093/nar/gkz517

**Published:** 2019-06-13

**Authors:** Kirill V Mikhailov, Boris D Efeykin, Alexander Y Panchin, Dmitry A Knorre, Maria D Logacheva, Aleksey A Penin, Maria S Muntyan, Mikhail A Nikitin, Olga V Popova, Olga N Zanegina, Mikhail Y Vyssokikh, Sergei E Spiridonov, Vladimir V Aleoshin, Yuri V Panchin

**Affiliations:** 1 Belozersky Institute of Physico-Chemical Biology, Lomonosov Moscow State University, Leninskiye Gory 1-40, Moscow 119991, Russian Federation; 2 Kharkevich Institute for Information Transmission Problems, Russian Academy of Sciences, Moscow 127994, Russian Federation; 3 Severtsov Institute of Ecology and Evolution, Moscow 119071, Russian Federation; 4 Institute of Molecular Medicine, Sechenov First Moscow State Medical University, Moscow 119991, Russian Federation; 5 Center for Data-Intensive Biomedicine and Biotechnology, Skolkovo Institute of Science and Technology, Moscow 143028, Russian Federation

## Abstract

Inverted repeats are common DNA elements, but they rarely overlap with protein-coding sequences due to the ensuing conflict with the structure and function of the encoded protein. We discovered numerous perfect inverted repeats of considerable length (up to 284 bp) embedded within the protein-coding genes in mitochondrial genomes of four Nematomorpha species. Strikingly, both arms of the inverted repeats encode conserved regions of the amino acid sequence. We confirmed enzymatic activity of the respiratory complex I encoded by inverted repeat-containing genes. The nucleotide composition of inverted repeats suggests strong selection at the amino acid level in these regions. We conclude that the inverted repeat-containing genes are transcribed and translated into functional proteins. The survey of available mitochondrial genomes reveals that several other organisms possess similar albeit shorter embedded repeats. Mitochondrial genomes of Nematomorpha demonstrate an extraordinary evolutionary compromise where protein function and stringent secondary structure elements within the coding regions are preserved simultaneously.

## INTRODUCTION

Inverted repeats enabling hairpin formation in nucleic acid sequences are known to function in a variety of biological processes. Hairpin structures provide sites for interaction with proteins and play important roles in cellular processes such as initiation of replication, regulation of transcription, recombination and DNA packaging (1,2). The hairpins rely on base pairing between the complementary arms of comprising inverted repeats to maintain their structure and fulfil their biological function. They are commonly associated with non-coding DNA and rarely overlap with the coding sequences, which are subject to selective constraints for functional gene products and optimal codon usage (3,4). Although the degeneracy of the genetic code has the capacity to accommodate secondary structure elements within protein-coding sequences, the protein-level and nucleotide-level selection limits the options for incorporation of highly structured elements.

There are only a few reported examples of stable secondary structures occurring within the coding sequence, and they usually involve specific insertion elements. One example is the transposable palindromic elements found in some *Rickettsia* species (5,6). Insertions of these palindromic elements do not abrogate enzymatic activity of the encoded proteins, allowing them to exist within the coding sequence (7). The stem–loop element required for selenocysteine inclusion—the selenocysteine insertion sequence (SECIS)—was reported to be incorporated in the protein-coding sequence of the fowlpox virus glutathione peroxidase without compromising the function of the enzyme (8). Among other examples are tRNA genes, which are partially or fully integrated within mitochondrial protein-coding genes of woodlice and presumably require alternative processing for the production of either a complete mRNA or a complete tRNA (9). The attenuator structures found in some bacterial amino acid biosynthesis and antibiotic resistance operons rely on pairing of inverted repeats (10,11). The attenuators consist of three repeats capable of forming alternative hairpin structures, and are embedded in the DNA sequence encoding the leader peptide (12). However, the leader peptide of the attenuators is usually encoded only by one arm of the inverted repeat, and the translated sequence has no established role in the cell. An exceptional case is the Rev response element (RRE)—a highly structured and conserved element appearing in lentiviral genomes that overlaps the coding region of env gene and is required for interaction with the viral protein Rev (13).

Whenever the secondary structure elements naturally occur within the coding gene regions, the effect of these elements on the sequence is subtle. Several genes in vertebrates were found to harbor conserved nucleotide secondary structure elements in the coding regions that might be involved in regulating the efficiency of splicing (14). These structures are comprised of multiple short and non-perfect duplex regions. Furthermore, it was shown that the protein-coding regions of mRNAs in general demonstrate greater negative free energies of folding than random sequences of the same length and nucleotide composition (15,16).

Here we report on the remarkable case of palindromic elements coexisting with functional genes, which challenges modern understanding of the constraints imposed on coding sequences by secondary structure elements within them. We sequenced the mitochondrial genomes of four Nematomorpha species and discovered perfect inverted repeats of unprecedented length (up to 142 bp in each arm) embedded within their protein-coding sequences. Nematomorpha or horsehair worms are an ecdysozoan phylum of parasitoid organisms that develop as endoparasites of arthropods, and are free-living as adults (17). Until now, Nematomorpha has been among the few invertebrate phyla for which there were no data on mitochondrial genomes. The extensive secondary structure elements are known to interfere with DNA sequencing (18). In order to overcome this difficulty and confirm the existence of inverted repeats in nematomorph sequences, we applied the enrichment procedure for palindromic DNA. We propose that these repeats likely represent a novel type of non-transmissive parasitic DNA elements or DNA disorders, and find rare examples of similar although shorter and less prevalent structures occurring in other mtDNA sequences.

## MATERIALS AND METHODS

### Sample sources and description

The specimens of *Gordionus alpestris* (Villot, 1885) were collected by Vladimir Yu. Schmatko and Boris D. Efeykin from the Syuk stream near the Nickel’ settlement in Republic of Adygeya (44°10′40.1″N 40°09′28.2″E) during the summer seasons of 2012–2016. The natural host of these nematomorphs in the collection area is a diplopod *Pachyiulus krivolutskyi* (Golovatch, 1977). Several specimens of *G. alpestris* were obtained after capturing the diplopods from the collection area and transporting them to the laboratory. The primary identification of hairworm species was based on morphological features and later confirmed by the analysis of 18S and 28S rDNA sequences. The 18S rDNA sequence was deposited in NCBI GenBank under accession number KT202292.

The male specimen of *Gordionus wolterstorffii* (Camerano, 1888) was collected by Boris D. Efeykin from a small stream near the Zvenigorod Biological Station of the Moscow State University in June 2014. The stream is a right tributary of the Moscow river (55°39″34.7′N 36°39′11.3″E). The obtained sequence of 18S rDNA was found to be close to the deposited 18S rDNA sequence of *G. wolterstorffii* from Germany (AF421765). The difference between these two sequences was only four substitutions in the gene of 1738 bp. The 18S rDNA sequence of the collected specimen was deposited in NCBI GenBank under accession number KT202294. The host of this specimen is unknown.

The male specimen of *Gordius sp*. was collected by Boris D. Efeykin from the ‘First Golden stream’ in the ‘Kedrovaya Pad’ Reserve in the ‘Land of the Leopard’ National park in Primorskii Region of the Russian Federation (43°05′50.8″N 131°33′32.9″E) in July 2014. The primary identification up to the genus was based on the morphology of the posterior end of the specimen, which demonstrated typical postcloacal crescent. The 18S rDNA sequence of this specimen was deposited in NCBI GenBank under accession number KT202295. Host of this specimen is unknown, but other *Gordius* specimens from the Kedrovaya Pad Reserve were observed leaving the body of a large orthopteran *Decticus verrucivorus*.

The female specimen of *Chordodes* sp. was presented by Dr A.V. Kompantzev, who collected this nematomorph in Sumatra, Indonesia in November 2014. Precise data of the collection site are absent. Primary identification of this specimen up to the genus level was based on morphological features: structure of the cuticle with numerous clusters of protruding areoles, including crowned ones. The 18S rDNA sequence of this specimen was deposited in NCBI GenBank under accession number MF113255. Host unknown.

### Sequencing and assembly of mitochondrial genomes

Sequencing of nematomorph mitochondrial genomes was performed with the Illumina platform. The sequencing libraries were prepared from total DNA extracts of individual worms following the TruSeq library preparation protocol. Paired-end libraries for *Chordodes* sp., *Gordius* sp. and *Gordionus wolterstorffii* were sequenced using the HiSeq system, generating from 10–20M 2 × 100 bp reads for each library. Two individuals of *G. alpestris* were sequenced separately—one using the MiSeq system, producing 9M read pairs with the read length of 250 bp, the other with the HiSeq system, producing 40M 2 × 100 bp reads. The mean insert sizes in the sequenced libraries are estimated to be in the range of 240–300 bp with standard deviations of 60–120 bp.

The assemblies were performed with SPAdes (19) after trimming the adapter sequences from the read data with Trimmomatic (20). Mitochondrial contigs were detected in the assemblies using BLAST searches (21) with the standard set of metazoan mitochondrial-encoded protein sequences. Invertebrate mitochondrial code was confirmed for these nematomorph species by both GenDecoder v1.6 (22) and FACIL (23). In all four nematomorph species, the automatic assembly resulted in fragmented mitochondrial contigs—the sequences were broken up primarily in the protein coding gene regions containing inverted repeats. The fragmented mitochondrial sequences were extracted from the assemblies and joined manually by aligning reads to the ends of contigs and extending the contig sequences until sufficient overlap between contigs was achieved. The reconstructed mitochondrial contigs were screened for assembly artifacts by mapping the read data with bowtie2 (24) and inspecting the alignments visually using Tablet alignment viewer (25).

### Inverted repeat enrichment

Experiments with DNA denaturation-renaturation and nuclease digestion were used for enrichment of DNA samples with palindrome sequences to confirm the validity of inverted repeats in assemblies. Extracted DNAs of the four nematomorph species were incubated for 5 min at 95°C to prepare single-stranded DNA and then immediately placed on ice. About 50 ng of denatured DNA was mixed with 1 U Mung Bean nuclease (SibEnzyme, Russia) and incubated for 15 min at 30°C. The digestion time was determined by PCR amplification of ribosomal RNA genes: the samples were considered to be sufficiently digested when no products were obtained in the PCR. The nuclease was inactivated by adding Tris-acetate, pH 8.3. The libraries for Mung Bean nuclease-digested samples of nematomorphs were constructed using TruSeq library preparation protocol (Illumina) and sequenced on a HiSeq 2000 instrument. From 5.1–17.1M paired-end reads were obtained for each library. The reads were mapped to the mitochondrial genome assemblies with bowtie2 (24) using very sensitive-local alignment mode.

### RNA-Seq analysis

To confirm transcription of the inverted repeat-containing genes and to examine the possibility of repeats impacting the gene expression in the host-borne and swimming stages of hairworms, we have collected several specimens of *G. alpestris* for RNA-Seq. Total RNA extracts from individual specimens were obtained using TRIzol reagent lysis followed by purification with the RNeasy kit (QIAGEN). The sequencing libraries were prepared from total RNA extracts using TruSeq RNA library preparation kit and sequenced with Illumina HiSeq and NextSeq systems. The libraries for differential expression analysis were constructed using the stranded version of the protocol without poly-A selection. We obtained 46M read pairs (100 bp reads) for a library from one individual of *G. alpestris*, and from 23–31M read pairs (150 bp reads) for each of the six libraries prepared for the differential expression analysis: 3 libraries were constructed for specimens that were extracted from the host, and another 3 for specimens captured in the water. The reads were trimmed with Trimmomatic (20) and mapped to the mitochondrial genome assembly of *G. alpestris* with bowtie2 (24). The per gene read counts were calculated with the BEDTools multicov utility (26). Analysis of the expression data for the libraries from the host-borne and swimming hairworm stages was performed with the edgeR package (27) in the R software (28) using the exact test.

### Inverted repeat analyses

To catalog the unusual inverted repeat structures observed in the mitochondrial genomes, we employed the repeat detection programs repfind (29) of the GenomeTools package (30) and einverted of the EMBOSS package (31). The repfind program reports maximal perfect repeats in the sequences, while the einverted program allows detection of imperfect repeats. We used the default scoring scheme for repeat detection with einverted (match 3; mismatch −4; gap penalty 12) and the score threshold of 15. For the eukaryote-wide analysis of inverted repeats in the mitochondrial coding sequences, we extracted the corresponding sequence records from the NCBI organelle genome resource (32) using the Entrez Direct efetch utility. A total of 132 281 coding sequences from 8952 mitochondrial genomes were collected and analyzed with the repfind program. To avoid repeat detection across sequences, the repfind program was ran for each coding sequence individually.

The relative positions of inverted repeats in genes were analyzed by constructing alignments of coding sequences using the TranslatorX (33) and MAFFT (34) alignment programs. For the construction of sequence conservation profiles, we selected a varied set of 46 mitochondrial genomes of protostome animals, including the 4 genomes of Nematomorpha. The alignments of amino acid sequences of protein-coding genes were prepared with MAFFT and concatenated by SCaFoS (35) for standardized evaluation of relative site rates. The site-wise evolutionary rate estimates for the concatenate were computed with IQ-TREE (36) using the mtZOA+F+R8 evolutionary model, selected as best fit by the built-in ModelFinder (37). For illustrations of conservation profiles, a smoothing function was applied to the site rate estimates using the central moving average with a window of size 5.

Compositions of amino acid sequences coded by the inverted repeats and non-repeat regions were compared using the Composition Profiler (38). Statistical significance of differences was evaluated using Fisher’s exact test with Bonferroni correction implemented in the Composition Profiler. Differences with alpha value below 0.05 (*P*-value < 0.0025) were considered significant.

The inverted repeats in coding sequences were classified in accordance to the relative positions of codons in the complementary arms of the repeat. The repeats with complementary codon positions 1 were designated as phase 1 repeats, repeats with complementary codon positions 2 as phase 2 repeats and repeats with complementary codon positions 3 as phase 3 repeats. We have computed the average number of amino acids that the repeats of each phase can accommodate given a fixed amino acid sequence coded by the complementary arm of the repeat. The measure of permitted amino acids was computed on the basis of the observed dipeptide frequencies in the entire set of nematomorph coding sequences, as follows. For each dipeptide, we enumerated the number of possible dicodons and the corresponding complementary codons that the repeat of each phase can encode. We counted the number of different amino acids for the complementary codons using the appropriate genetic code (invertebrate mitochondrial code). The weighted average of the number of possible different amino acids was then obtained by summing over the dipeptide frequencies. The mean entropy calculation utilized the same procedure, except instead of counting the possible complementary amino acids for a dipeptide, their entropy was computed using either the actual codon frequencies or equal codon frequencies.

For estimation of the expected inverted repeat counts and the relative repeat phase proportions, we applied a sequence randomization procedure. The mitochondrial coding sequences were randomized by shuffling codons, thus preserving the original codon frequencies. The shuffling was performed using the uShuffle (39) implementation of the MEME suite (40). We used the kmer 1 setting of uShuffle after recoding all codons with a single character code. Ten replicates were constructed with random seeds for the analysis of the expected inverted repeat counts. For the analysis of repeat phase proportions, a replicate with shuffled codons and a replicate with shuffled nucleotides (kmer 1 setting without single character encoding) were created.

### 18S rRNA gene phylogeny

To determine the phylogenetic relationship between the sequenced species and how broadly they sample the nematomorph diversity, we reconstructed the phylogeny using the available 18S rRNA genes. The 18S rRNA genes of the four sequenced nematomorph species were aligned with other nematomorph and ecdysozoan sequences in the NCBI GenBank database (https://www.ncbi.nlm.nih.gov/genbank/). Four sequences from Lophotrochozoa were added as an outgroup for phylogenetic analysis. The alignment was done with MAFFT (34) and revised manually in BioEdit (41). Ambiguously aligned regions were excluded from the analysis, producing a 1496 nucleotide position alignment. The phylogeny was reconstructed using the Bayesian inference approach with MrBayes (42) under the GTR substitution model with eight categories of Gamma-distributed rates, estimation of invariable site proportion and a covarion model. The consensus tree was generated from four independent runs of four Metropolis-coupled Markov chains that were sampled across 10 million generations and summarized with a 50% burn-in.

### Worm tissue preparation for respirometry experiments

Each individual worm was separated from its external cuticle by squeezing worm tissues out from the cuticle using a steel spatula at +4°C. Immediately thereafter the worm tissues were placed in an individual precooled freezing-vial of 1.5 ml volume supplied with 0.2 ml medium A containing 20 mM Tris-HCl (pH 7.3), 0.25 M sucrose, 0.2 mM EGTA, 0.2 mg/ml bovine serum albumin essentially fatty acid-free and 0.17 mM PMSF and quick-frozen in liquid N_2_. All the prepared worm tissue samples were kept at −75°C for long-term storage.

The respiratory activity measurements were performed with the mitochondria-containing samples prepared by homogenization of worm tissues. Before homogenization, a frozen tissue sample of each individual worm was thawed out in an ice bath. Then, 0.5 ml of cold medium A was added and the mixture was placed into a precooled glass–glass conical tissue grinder. Initially, the mixture was briefly homogenized manually with a smooth glass pistil and thereafter the procedure was continued using an electric mixer with a glass pistil (revolving speed 800 rpm). Homogenization was performed in ten 10 s periods with cooling of the glass conical tissue grinder in an ice bath between homogenization periods. After that the obtained tissue suspension was centrifuged (500 rpm, 5 min, 4°C, Eppendorf centrifuge, rotor F-45) to remove undisrupted tissue bits and the supernatant was sedimented to obtain mitochondria-containing pellet (13500 rpm, 15 min, 4°C, Eppendorf centrifuge, rotor F-45). The obtained pellet was homogenized in a Potter mini-glass-homogenizer manually and freeze-thawed three times to cause the disruption of mitochondrial membranes (43). The homogenate was frozen in aliquots at −70°C until measurements.

### Respiratory activity measurements

The oxygen consumption in the horsehair worm tissue homogenate suspensions was determined polarographically using an Oxygraph Plus System containing the thermostatically controlled chamber with a Clark-type oxygen electrode (Hansatech Instruments, England) connected to PC. The digital signals incoming from Oxygraph System to PC were recorded with the software ‘O_2_View’ supplied by the manufacturer. All the measurements were performed in the sample volume of 250 μl under vigorous stirring at 37°C; the incubation medium contained 20 mM Mes/Tris (pH 6.0), 2.5 mM MgSO_4_, 0.2 mM EGTA, antibiotic alamethicin 50 μg/ml and 200 μM NADH. The oxygen consumption was started by the addition of tissue homogenate sample. The measurements were performed in three biological replicas, i.e. with three different tissue suspensions.

## RESULTS

**Figure 1.**
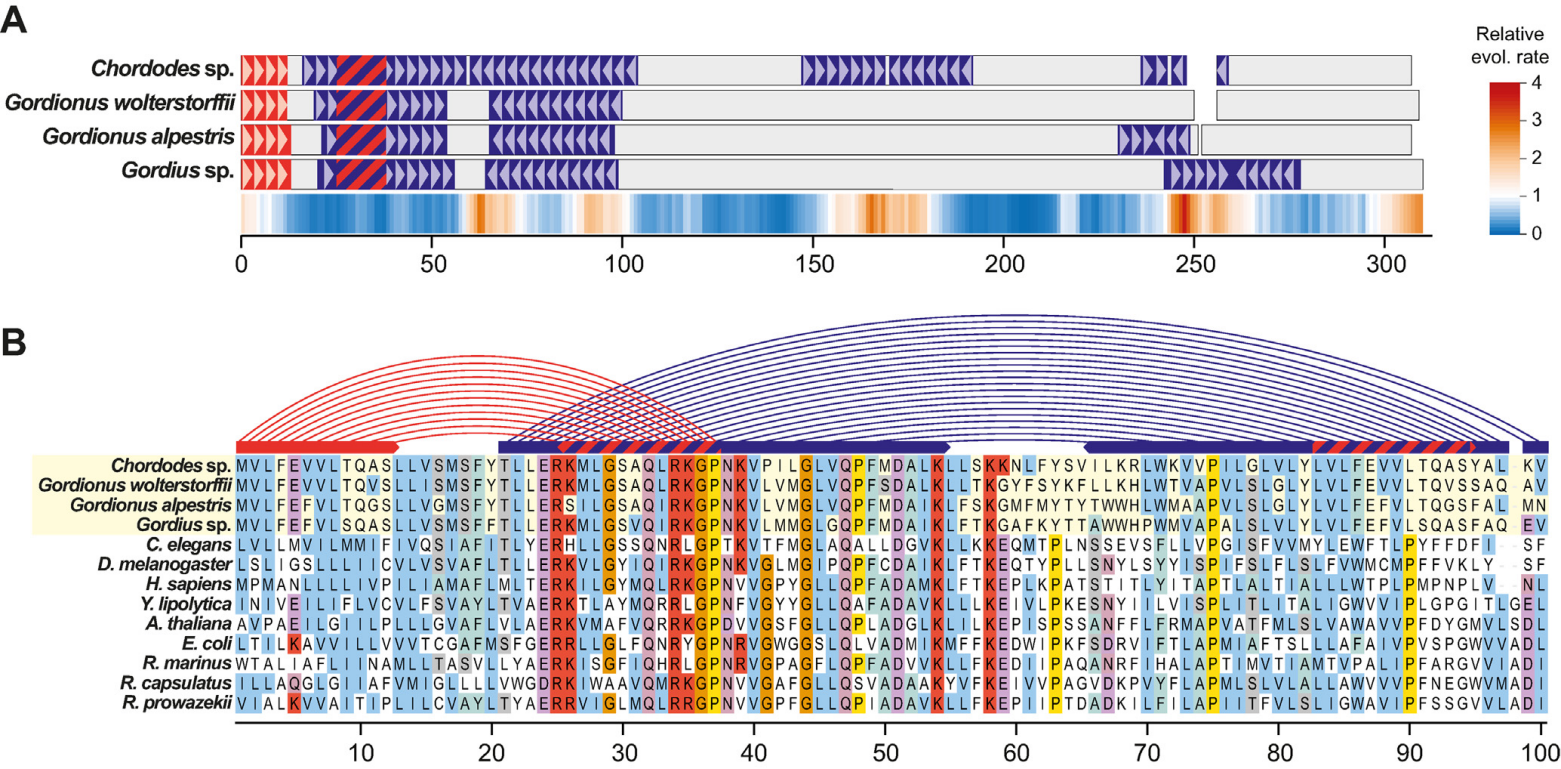
Inverted repeats in the coding sequences of nad1 genes. (**A**) Schematic depiction of an amino acid alignment of nad1 sequences from four species of Nematomorpha; inverted repeats in the underlying nucleotide sequences are highlighted in purple or orange, regions of overlapping repeats are indicated with a striped pattern; a conservation profile of nad1, derived from an amino acid alignment of animal sequences (Materials and Methods section), is depicted below the alignment as an estimate of site rates. (**B**) A portion of the nad1 amino acid sequence alignment (sites 1–100) showing conservation of amino acids encoded by both arms of the inverted repeat in nematomorph sequences; the major inverted repeat (purple) and the shorter overlapping repeat (orange) are shown schematically above the alignment; the overlapping region of the repeats and the resulting direct repeat are indicated with a striped pattern.

Using the Illumina platform, we sequenced the mitochondrial genomes of four Nematomorpha species: *Gordius* sp., *Gordionus wolterstorffii, Gordionus alpestris* and *Chordodes* sp. The assembled mitochondrial genomes have similar sizes (∼15 kb) and gene orders, and encode a set of 13 proteins, ribosomal RNAs and transfer RNAs (Supplementary Figure S1), which are typical for mitochondrial genomes of animals (44). The A+T content of the sequenced genomes ranges from ∼65% in the *Gordionus* species to 78% in *Chordodes* sp. (Supplementary Tables S1 and S2). An extraordinary feature of all four nematomorph mitochondrial genomes is the presence of perfect inverted repeats within conserved regions of protein-coding sequences (Figure 1). In total, we detected 110 perfect inverted repeats of at least 15 bp in the four genomes. The mean length of repeats is 43 bp, with the largest repeat (142 bp) forming a perfect palindrome of 284 bp in the nad6 gene of *Gordius* sp. The close positioning of inverted sequences suggests that they form hairpin structures *in vivo*: the average distance between the inverted sequences is 37 bp. At least 48 inverted repeats appear to be species-specific: their locations in the coding sequences are not shared with other species. However, several longer repeats tend to share midpoint positions in the sequence of two or more nematomorph mitochondrial genomes. Four inverted repeats were found in the same locations in all four species: an inverted repeat in atp6, nad1 and two inverted repeats in nad5. In several cases inverted repeats were found to overlap, corresponding to a putative complex with competing hairpins and direct repeats, reminiscent of the bacterial attenuator structure. Importantly, we found that both arms of the inverted repeats encode conserved amino acids (Figure 1B). This suggests that these regions are not processed but are translated into functional proteins.

**Figure 2.**
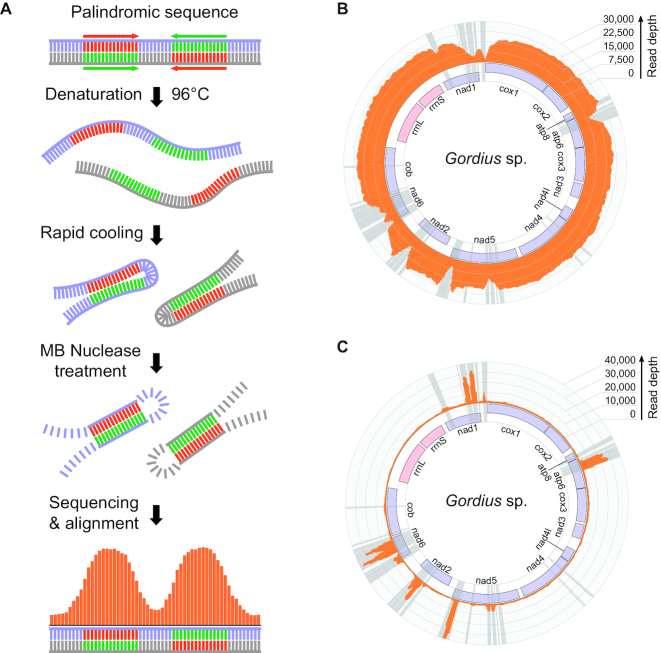
Inverted repeat enrichment experiment with the mitochondrial DNA of Nematomorpha. (**A**) The DNA digestion procedure employed for confirming the inverted repeat sequences, involving DNA denaturation, rapid renaturation, and treatment with a single-strand-specific mung bean nuclease. (**B**) Histogram of read depth over the mitochondrial genome of *Gordius* sp. with reads from a sequencing library of untreated DNA; inverted repeat regions are depicted in gray. (**C**) Mapping of reads from a library of nuclease-treated DNA, showing the enrichment of inverted repeat sequences.

Long inverted repeats are associated with drops in read coverage leading to problems with the assembly of repeat-containing regions. Therefore, we sought to confirm their existence and the validity of assemblies using an enrichment procedure for regions with secondary structure. We applied an approach similar to the GAP-seq method for detection of palindromic DNA (45). Total DNA extracts were diluted and subjected to rapid denaturation–renaturation followed by treatment with Mung bean nuclease to remove single-stranded DNA molecules. Palindromic sequences are at an advantage in reforming double-stranded DNA after denaturation over non-palindromic DNA, and are therefore more resistant to the nuclease treatment (Figure 2A). The sequencing of the nuclease-treated samples recovered the expected enrichment of sequences comprising predicted inverted repeats (Figure 2B and C). The enrichment procedure confirms that the palindromic sequences occur naturally in mitochondrial DNA of Nematomorpha.

The capability of inverted repeats to form hairpins and affect DNA replication was observed in a PCR amplification and restriction experiment. We designed a pair of primers to amplify a fragment of the *G. alpestris* nad6 gene that should produce a 615 bp product containing overlapping palindromic sequences according to the assembly (Supplementary Figure S2A). Instead, the PCR recovers shorter products missing the longest palindromic sequence. We used a restriction enzyme that cuts at a site present in the assembly but not in the amplified sequences. Consistent with the assembly based prediction, the HphI restriction enzyme treatment prevented product amplification. This suggests that the shorter products are not native to the mitochondrial sequence, and are likely a result of PCR jumping facilitated by the presence of repeats flanking the main hairpin and leading to the skip of its sequence (Supplementary Figure S2B and C). Absence of shorter products without the palindrome in the amplification reaction with the restrictase-treated sample also suggests that the DNA extracts from nematomorpha do not naturally contain detectable amounts of mtDNA variants where the hairpin sequence is lost.

**Figure 3.**
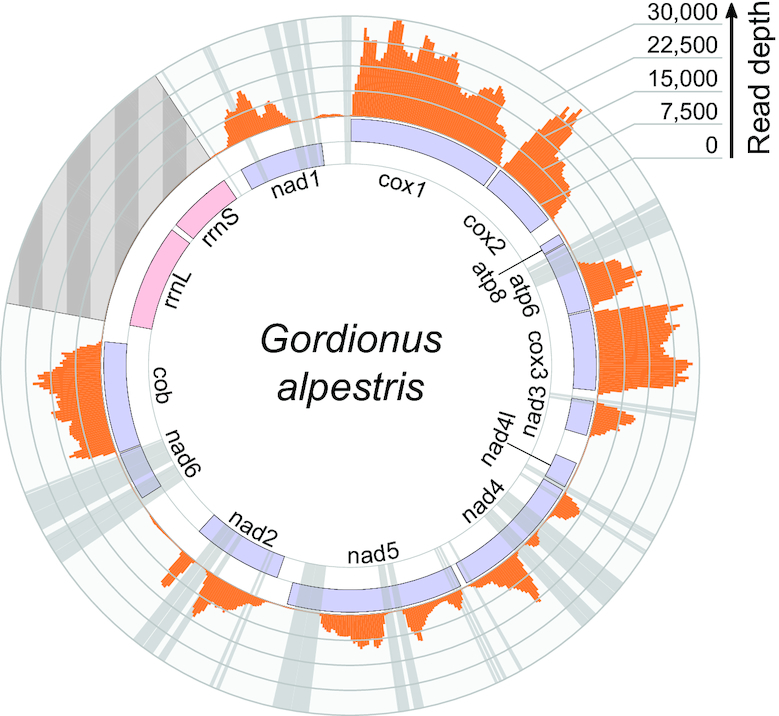
Mapping of reads from an RNA-Seq library of *Gordionus alpestris*. The histogram shows read depth over the mitochondrial genome of *G. alpestris* with the inverted repeat regions shaded gray; region containing the highly expressed rRNA genes was masked during the mapping (indicated with a striped pattern).

The mapping of reads from an RNA-Seq library of *G. alpestris* confirmed that both the repeat-free and the repeat-containing mitochondrial genes are transcribed. The regions containing long inverted repeats experience drops in read depth, resulting in fragmentary coverage for the nad1, nad2, nad4, nad5 and atp6 genes and nearly complete masking of the repeat-heavy nad6 gene in *G. alpestris* (Figure 3). The uneven coverage of inverted repeat-containing genes is similarly observed in the DNA libraries (Figure 2B). This suggests that the coverage issues are a consequence of inverted repeats hindering the sequencing of corresponding transcript regions. Apart from the poor recovery of long inverted repeats, the RNA-Seq does not reveal any systematic differences between the cDNA and genomic sequences, indicating lack of wide-scale RNA editing in nematomorph mitochondria.

Using RNA-Seq, we explored the possibility of inverted repeats playing a role in regulating the intensity of respiration through differential mitochondrial gene expression between the host-borne and swimming stages of hairworms. We analyzed six specimens of *G. alpestris*, three of which were directly extracted from the diplopod hosts, and the other three captured free from hosts in water, and compared their levels of mitochondrial gene expression. The RNA-Seq from the six specimens further confirmed the transcription of the repeat-containing genes, but revealed no significant differences in the expression levels between the host-borne and swimming stages (Supplementary Table S3).

We have confirmed presence of the nad1 protein in the tissue homogenates of *G. alpestris* using PAAG/Western Blot with anti-human nad1 antibodies (Supplementary Figure S3A). We found that the oxygen consumption rate by *G. alpestris* muscle tissue homogenates stimulated by NADH can be abolished by rotenone, a specific inhibitor of respiratory complex I (Supplementary Figure S3B). This suggests that the respiratory chain in Nematomorpha is functional.

**Figure 4.**
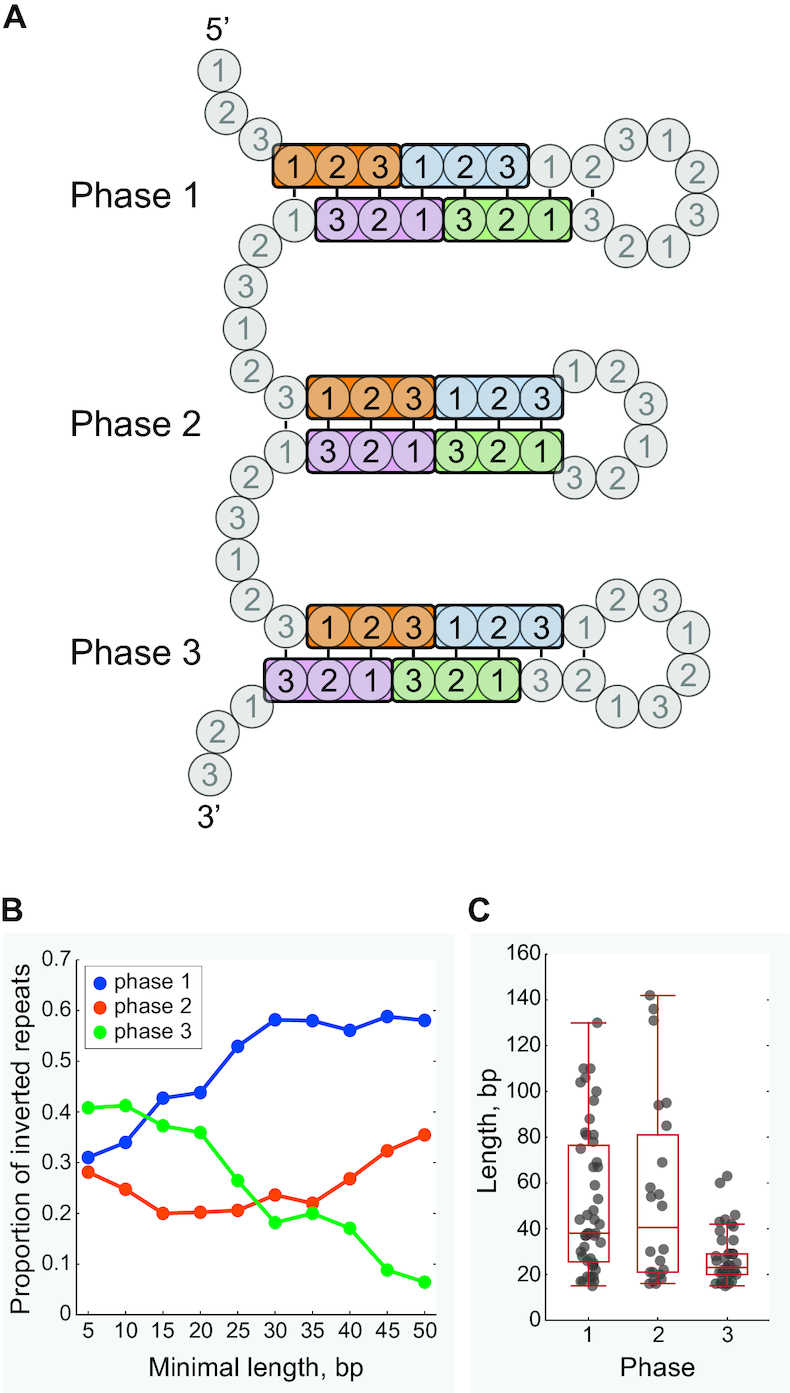
Characterization of the three inverted repeat phases for coding sequences. (**A**) Three possible relative arrangements of codons in the complementary arms of inverted repeats: phase 1 repeats tie together codon positions 1 between the complementary arms of the repeat, phase 2 repeats tie together codon positions 2 and phase 3 repeats tie together codon positions 3. (**B**) Dependence of the relative amounts of repeats of each phase on the minimal length of the repeat in the genomes of Nematomorpha; the total number of detected repeats drops from 35 487 at the minimal length of 5 bp to 68 at the minimal length of 25 bp, and to 31 at the minimal length of 50 bp. (**C**) Distributions of inverted repeat lengths by phase in nematomorph species (for repeats over 15 bp), showing individual repeat occurrences.

Amino acid sequences encoded by the complementary arms of an inverted repeat are codependent. The relative position of codons in the complementary arms determines the phase of the inverted repeat (Figure 4A). The mitochondrial genomes of Nematomorpha display shifts in the relative proportions of repeat phases in response to the minimal length of the repeat (Figure 4B). The preponderance of short inverted repeats with mutually complementary third codon positions (phase 3 repeats) appears to be largely guided by the codon composition of the coding sequences, and follows closely the phase distributions seen in other invertebrate mitochondria (Supplementary Figure S4A–D). The proportion of phase 3 repeats declines with increasing repeat length in nematomorph mitochondria, and repeats exceeding the length of 65 bp are represented exclusively by phase 1 and phase 2 repeats (Figure 4B and C). The observed depletion of phase 3 repeats might be linked to the strictness of constraints these repeats impose on the encoded sequences. By tying in first and second codon positions in the complementary sequences, phase 3 repeats impose the strongest restrictions on the diversity of amino acid sequences the repeat can accommodate under a fixed amino acid sequence encoded by the opposite arm of the repeat (Table 1). Phase 1 repeats, on the other hand, impose the weakest constraints by tying in the functionally definitive second codon position with the third. The cumulative length of repeats of each phase correlates negatively with the constraint strictness, and the correlation becomes more pronounced with increasing minimal repeat length (Table 1). This suggests that the differences in the constraining pressures of repeat phases play an important role in the emergence of long inverted repeats, and that these repeats are under selection acting to preserve the function of encoded proteins.

**Table 1.** Characteristics of inverted repeat phases

	Phase 1	Phase 2	Phase 3
Mean number of permitted amino acids	3.84	3.21	1.59
Mean entropy of amino acid states, bits	1.20	1.11	0.50
Mean entropy of amino acid states, bits (equal codon frequencies)	1.80	1.63	0.45
Observed number of repeats >15 bp	47	22	41
Observed cumulative length of repeats >15 bp	2419	1207	1120
Observed number of repeats >30 bp	32	13	10
Observed cumulative length of repeats >30 bp	2101	1030	448

The number of permitted amino acids denotes how many different amino acids can be accommodated by the reverse strand without changing the amino acids coded by the forward strand, averaged across the length of the sequence; entropy provides a measure of permitted amino acids with the account of naturally observed or equal codon frequencies (Materials and Methods section).

Repeat sequences of the three phases experience unequal biases in the codon usage and amino acid composition (Supplementary Text S1). All three types of repeat regions display more uniform synonymous codon usage than non-repeat regions: the effective number of codons (46) in the repeat regions is 64.4, 55.1 and 51.1 for the three phases, against 42.8 for the non-repeat regions. Phase 2 repeats, in particular, affect the hydrophobic amino acid composition in the palindromic regions. These repeats constrain the second codon positions to equal frequencies of complementary nucleotides. Accordingly, the frequencies of hydrophobic amino acids Leu, Phe, Ile, Val and Met, which all have T in the second codon position, were found to be decreased in phase 2 repeats (Supplementary Figure S5A–C). In a few cases, the repeat-bearing mitochondrial genes in Nematomorpha were found to contain insertions overlapping the repeat regions and encoding hydrophilic amino acids. Such insertions might not be essential for the protein function *per se* but are necessitated to accommodate the indispensable hydrophobic amino acids in the complementary arm of the inverted repeat.

**Figure 5.**
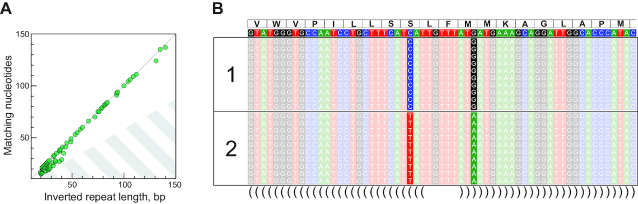
Concerted evolution of the complementary arms of inverted repeats in Nematomorpha. (**A**) Scatter plot of inverted repeat length versus number of matching sites for repeats discovered by einverted (Materials and Methods section), permitting imperfect match between the sequences; nematomorph repeats (depicted in green) are clustered on or near the diagonal; the striped region of the plot corresponds to the alignment scores below the allowed threshold under the default scoring scheme of einverted with a score cutoff of 15. (**B**) A portion of read alignments with libraries from two individuals of *Gordionus alpestris*, featuring the central region of a hairpin in nad2; the reference sequence (above) is based on the library of the first individual; translated sequence is given on top of the alignment; the hairpin arrangement is indicated below the alignment using the parenthesis notation (a fuller version of the alignments is given in Supplementary Figure S8).

Small subunit rRNA phylogeny places the sequenced hairworm species in distant branches of the nematomorph tree (Supplementary Figure S6). Therefore, the presence of inverted repeats in mitochondrial coding sequences is an ancestral feature of all freshwater species of Nematomorpha. Currently, no other members of Nematomorpha, including the early branching marine genus *Nectonema*, have any genomic data available for a fine-grained resolution of inverted repeat evolution or elucidation of their emergence. Longer inverted repeats tend to have common midpoint positions in several species, suggesting their inheritance from a common ancestor and permanence in nematomorph evolution (Supplementary Figure S7A–C). Shared repeats do not appear to preclude the mutability of underlying sequences—common repeat regions show interspecies variation in sequence and repeat length, while maintaining the identity between the arms of inverted repeats (Supplementary Figure S7D and E). We searched the nematomorph mitochondrial genomes for evidence of imperfect or decaying inverted repeats. Most of the discovered repeats over 30 bp display 100% identity (Figure 5A), and the few long repeats with lower sequence identity differ only by distal positions in the repeat or an indel. We also observe concerted evolution of inverted repeat sequences at smaller time scales—between individuals of the same species. We sequenced another specimen of *G. alpestris*, which revealed 24 substitutions in the mitochondrial DNA. Two substitutions were located in the repeat region of nad2 gene and correspond to the paired nucleotides of the complementary arms of the inverted repeat (Figure 5B and Supplementary Figure S8). The substitutions result in a non-synonymous change in the coding sequence but preserve the perfectness of the repeat.

**Figure 6.**
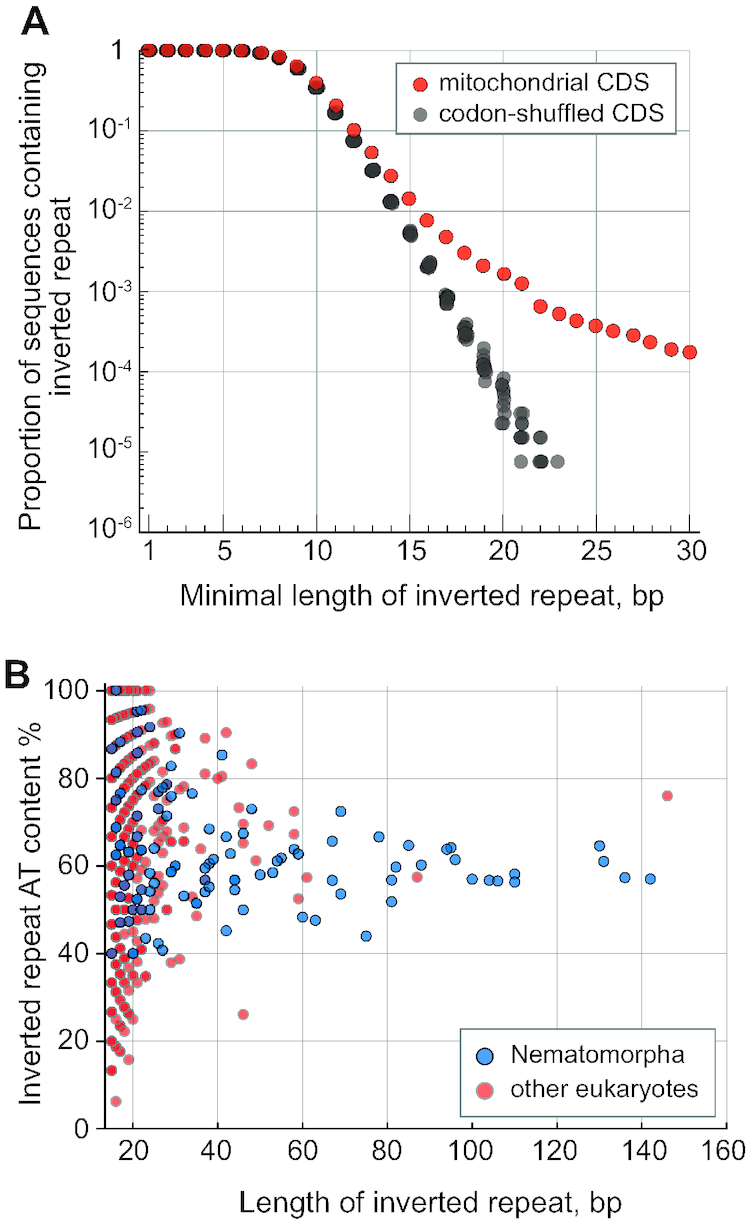
Characteristics of perfect inverted repeats in the protein-coding sequences of mitochondrial genomes. (**A**) Over-representation of inverted repeats in the mitochondrial coding sequences of eukaryotes (excluding Nematomorpha) (red) over the repeats in sequences with randomly shuffled codons—10 replicates (gray). (**B**) Plot of perfect inverted repeat length versus the repeat AT content in the mitochondrial coding sequences of Nematomorpha (blue) and other eukaryotes (red).

**Figure 7.**
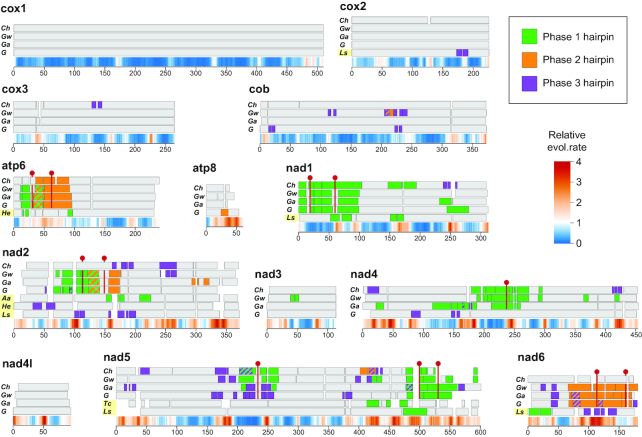
Schematic depiction of amino acid sequence alignments with mitochondrial genes of Nematomorpha (*Ch, Chordodes* sp.; *Gw*, *Gordionus wolterstorffii*; *Ga*, *Gordionus alpestris*; *G*, *Gordius* sp.), featuring repeat-containing genes of other invertebrates: *Ls*, *Lepidodermella squamata*; *Aa*, *Aleurochiton aceris*; *He*, *Hoploplana elisabelloi*; *Tc*, *Thaumamermis cosgrovei*. The positions of inverted repeats in sequences are marked with colors corresponding to the three inverted repeat phases, and the regions of overlap between repeats of different phases are marked with a striped pattern. Red pins mark midpoint positions of hairpins that are shared by at least three species. Each alignment is supplemented with a conservation profile corresponding to the site rate estimates inferred using a concatenate of amino acid alignments of animal sequences (Materials and Methods section).

To determine whether the phenomenon of long inverted repeats in mitochondrial coding sequences is unique to Nematomorpha, we analyzed the available mitochondrial genomes in the NCBI organelle genome database. Mitochondrial coding sequences display over-representation of perfect inverted repeats over random sequences utilizing the same codon frequencies (Figure 6A). The over-representation shows exponential dependence on the repeat length, which agrees with the repeat enrichment observed in non-mitochondrial and non-coding genomic data (47). We found 194 perfect inverted repeats exceeding 20 bp in a diverse set of eukaryotic mitochondrial genomes (Supplementary Table S4), whereas <10 such occurrences were expected by chance. Many of the identified repeats have highly biased AT content: in approximately half of the repeats the AT content exceeds 80%. By contrast, the long inverted repeats in Nematomorpha do not display high AT content (Figure 6B). Inverted repeats with characteristics similar to long repeats from Nematomorpha (AT content <80%, length >30 bp) are present in the mitochondrial coding sequences of other eukaryotes. The longest perfect inverted repeat (146 bp) is found in an uncharacterized ORF from a heterolobosean flagellate *Pharyngomonas kirbyi*. Another long repeat (87 bp) is found in an ORF from a myxosporean animal *Kudoa iwatai*. Both of these repeat instances, however, could not be verified through homology, and might be results of annotation errors. Barring the unconfirmed repeat findings, the most conspicuous case outside of Nematomorpha includes the gastrotrich *Lepidodermella squamata*, where repeats of up to 60 bp are found in the nad1, nad2, nad5, nad6 and cox2 genes (Figure 7). Less abundant or singular instances of long repeats are also found in other invertebrates: in the nad2 genes of hemipteran *Aleurochiton aceris* and flatworm *Hoploplana elisabelloi*, and in several genes from arthropods and nematodes (Supplementary Table S4). The sparse phylogenetic distribution of repeats and difference in the relative positions within genes rules out any possibility of their common origin. These occurrences indicate that the emergence of long inverted repeats in mitochondrial coding sequences is not an entirely unique phenomenon, but reaches an exceptional scale in Nematomorpha.

One more observation is apparent from the repeat occurrences: the relatively more conserved mitochondrial cytochrome oxidase genes are less likely to incorporate long repeat elements. This appears to hold for occurrences in Nematomorpha and other eukaryotes as well (Figure 7 and Supplementary Table S4): long repeats tend to emerge in the mitochondrial NADH dehydrogenase genes, but are never encountered in the conserved cox1 gene. Unexpectedly, the inverted repeats in the NADH dehydrogenase genes are not restricted to the highly variable regions, and are frequently found to overlap relatively conserved portions of the sequence with at least one arm of the repeat. This suggests that while the incorporation of repeats might depend on the sequence variability, highly variable gene regions could be detrimental to the survival of repeats in the sequence.

## DISCUSSION

The inverted repeats in the mitochondrial genes of Nematomorpha appear to be distinct from all other protein-coding secondary structure elements. The length of the repeats and lack of mismatches suggest that they are actively maintained throughout evolution. By contrast, the selfish palindromic elements of *Rickettsia* species were found to be losing repeat identity over time (7). The evolution of nematomorph hairpin structures is also dynamic—they appear scattered throughout the mitochondrial genes and can be lost or gained by genes of different species, which is at odds with their possible function as attenuating structures. The perfectness of the repeats also distinguishes them from the coding mRNA structures reported by Meyer and Miklos (14).

Although there are numerous examples of palindromic sequences in mtDNA, they are primarily restricted to non-coding regions. Many invertebrate species harbor inverted repeats in the AT rich control regions of their mtDNA (48). An unusual case of palindromic elements was also found in the green algae *Polytomella magna*, where every mitochondrial gene is present as a palindromic unit encoding two whole copies of a gene (49). In another green alga, *Lobosphaera incisa*, the mitochondrial genome carries non-coding repetitive palindromic sequences that might be involved in mediating genome rearrangements and gene expression (50). Also, baker’s yeast palindromic sequences can emerge as a result of recombination-mediated deletions of mtDNA sequences (51). Finally, protein-coding genes in mitochondria of yeast *Magnusiomyces capitatus* were found to be interrupted by multiple palindromic elements that are transcribed but remain untranslated due to translational bypassing (52).

Limitations imposed by inverted repeats in protein-coding genes are similar to those imposed by overlapping genes. In both cases, forward and reverse complement strands encode amino acid sequence. Overlapping genes are common in viruses, due to their constraints on the genome size (53,54), and there are also examples of antisense coding-genes in cellular organisms (55–57). Moreover, several mitochondrial genes encode short peptides in opposite reading frames (58,59). Finally, the short regions of variable ATP6 and ATP8 genes in human mtDNA partially overlap with each other (60). However, in viruses, bacteria and mitochondria overlapping genes have evolved either as a result of genome size reduction or as a mechanism of gene coregulation (61). By contrast, inverted repeats in Nematomorpha neither decrease the length of the genes they appear in nor provide any apparent options for coregulation.

It is not clear whether these repeats play any functional role in the mitochondria of Nematomorpha. The presence of secondary structure elements in the mRNA could potentially change its lifespan in the cells or regulate the translation rate and the fidelity of protein synthesis. Given that long palindromes do not appear in the cytochrome *c* oxidase genes of all four studied species, we cannot exclude the possibility that the repeats play a role in differential expression of respiratory chain complexes. The activities of respiratory chain complexes are usually coupled to each other (62), although in some cases organisms can benefit from differential expression of respiratory chain subunits. For example, the nematodes *Ascaris* can utilize fumarate as terminal electron acceptor (63) and, therefore, do not require cytochrome oxidase for maintaining the mitochondrial transmembrane potential. Also, the repression of NADH dehydrogenase expression level was shown to be an adaptive response to hypoxia (64). The RNA-Seq analysis of host-borne and swimming stages of hairworms revealed no significant differences in the expression levels of repeat-containing and repeat-free genes at the level of transcripts. Still, the regulatory function of palindromic sequences remains a possibility but might be restricted to certain cell types or larval stages or to mechanisms of translational regulation.

Another possibility for the nature of these inverted repeats is suggested by the binary character of repeat identity. We observe that nearly all long repeats display perfect match between the complementary arms—there is no excess of imperfect repeats, which would inevitably arise from the ‘tug-of-war’ between selective forces for conservation of encoded sequence and secondary structure. This suggests that the repeat identity is actively maintained by eliminating any mismatches as they appear. The existence of a repeat conversion mechanism would alleviate the necessity to postulate intrinsic functional significance of inverted repeats. Non-specific repeat conversion would likely counteract the loss of repeats irrespective of their functional role, ultimately giving them parasitic character. Such mechanism of repeat survival is especially relevant to repeats appearing in the coding sequences, because the alternative pathway of inverted repeat loss through deletion of the whole hairpin sequence is inaccessible in these cases. The observed dynamic nature of repeat evolution with multiple gains and losses also indicates that the repeats are not indispensable and their emergence is likely governed by a stochastic process.

What could be the mechanism for the formation and stability of palindromes in the mitochondrial genomes of Nematomorpha? Active maintenance of inverted repeat structures has been documented previously in the bacterial genomes (65), in the human X and Y chromosomes (66,67) and in the genomes of *Caenorhabditis* species (68). One possible mechanism for palindrome formation and concerted evolution of its complementary arms is the template switching mechanism (69). Template switches in the replication fork are associated with generation of perfect inverted repeats from imperfect quasi-palindromes, and are thought to be responsible for the over-representation of short inverted repeats in genomes (70). Longer inverted repeats might also be subject to conversion through the repair and recombination machineries. The mtDNA supercoiling could be a factor in mediating the formation of local hairpin structures with inverted repeats, exposing them to the repair machinery. Additionally, hairpin structures can form in single-stranded replication intermediates, which arise in asynchronous mtDNA replication models (71). None of these mechanisms, however, are specific to Nematomorpha, and cannot sufficiently explain the exceptional prevalence of inverted repeats in their genomes. It is also unclear how nematomorphs cope with the genomic instability commonly associated with the inverted repeats (72) given the exceptional length and abundance of these elements.

The derived state of nematomorph sequences, which is also noticeable in the rRNA gene phylogeny, suggests that they experienced accelerated evolution in their early history. We hypothesize that some aspect of the mtDNA maintenance in Nematomorpha conferring them susceptibility to inverted repeat accumulation was fixed under the conditions of weakened selection accompanying the phase of accelerated evolution. The observed prevalence of repeats might, therefore, be an evolutionary burden of the supposed mtDNA maintenance peculiarity rather than a functionally essential feature of their genomes.

The enigmatic phylum Nematomorpha has brought forth an evolutionary curiosity that reveals remarkable plasticity of the mitochondrial protein-coding sequences. Data from nematomorphs exemplify the utilization of genetic code degeneracy to optimize base pairing in the coding secondary structures. The observed unequal proportions of repeat phases confirm the predicted favorability of phase 1 pairings in the coding regions and the bias against phase 3 pairings (73). The dynamic nature of inverted repeats obscures their interpretation as functionally important elements of coding sequences, raising the possibility that they act akin to parasitic DNA elements, exploiting putative idiosyncrasies of mtDNA maintenance in Nematomorpha to continually preserve the identity between complementary repeat arms. While the mechanism generating and preserving the repeats in Nematomorpha remains to be established, the presence of similar albeit less conspicuous repeat elements in other mitochondrial genomes points to the existence of a possible common basis for this type of genome aberrations.

## DATA AVAILABILITY

The assembled mitochondrial genomes are deposited in NCBI GenBank under accessions MG257764-MG257767.

## Supplementary Material

gkz517_Supplemental_File
