# Supplementary material for: Coding palindromes in mitochondrial genes of Nematomorpha
Source: Nucleic Acids Res. 2019 Jun 13;47(13):6858–70. doi: 10.1093/nar/gkz517 (PMC6649704; doi:10.1093/nar/gkz517)
Supplement: gkz517_Supplemental_File [file gkz517_supplemental_file.pdf]

Text S1.

Nucleotide and amino acid contents in the inverted repeat regions of nematomorph mtDNA genes are affected by the constraints of the repeats. The three inverted repeat phases (phase 1: complementary codon positions 1-1, 2-3; phase 2: complementary codon positions 2-2, 1-3; phase 3: complementary codon positions 3-3, 1-2) show different biases in the nucleotide and amino acid compositions. One of the primary inherent biases of the mitochondrial encoded proteins – enrichment in hydrophobic amino acids and the consequent excess of thymine in the 2nd codon position, results in different outcomes for the compositions in the three types of repeats. In phase 1 repeats amino acids with codons NNY (Phe, Ile, Tyr, Asn) are significantly underrepresented, while chemically similar amino acids with codons NNR (Leu, Val, Trp, Gln) are overrepresented due to the high A/T ratio in 3rd codon position, which accommodates the low A/T ratio in the complementary 2nd codon position (Supplementary Figure S5). Phase 2 repeats cannot maintain high T content in the 2nd codon positions, as it requires equally high A content in this position, disrupting the hydrophobicity of the encoded regions. Accordingly, the amino acids with NTN codons are underrepresented in the phase 2 repeats (significantly for Leu, Ile, Met), while the glutamate codons GAR show enrichment in these repeats. Leucine codons CTA and TTA are absent in phase 2 repeats because they are complementary to the stop codons TAG and TAA. Proline CCG, threonine ACG, and alanine GCG codons complementary to the rare arginine codons are underrepresented (Supplementary Figure S5). Phase 3 repeats impose the strongest constraints on the ability of complementary sequences to accommodate diverse amino acids. These hairpins can partially accommodate the excess of T in the 2nd codon position by increasing the frequencies of ANN codons and decreasing the frequencies of TNN. This pattern of codon usage may lead to overrepresentation of Ile, Met, Thr, Asn, Lys and underrepresentation of Phe, Tyr, Trp and Cys. We observe significant underrepresentation of Tyr, but the amino acids Ala and Glu are overrepresented in these types of repeats (Supplementary Figure S5). Additional reason for the decreased Tyr content is the partial complementarity of its TAY codons to stop codons. Overrepresentation of glutamate codons GAR in phase 3 repeats may be a consequence of them being interlocked with the abundant serine codons TCN. Alanine codons GCN, however, are complementary to themselves. A possible explanation for alanine abundance in phase 3 repeats is that alanine is relatively “harmless”, i.e. can be incorporated without disrupting the protein fold. Therefore, Ala may be favored in regions where it is difficult to satisfy the constraints of protein structure by any amino acid pair with complementary 1-2 codon positions.

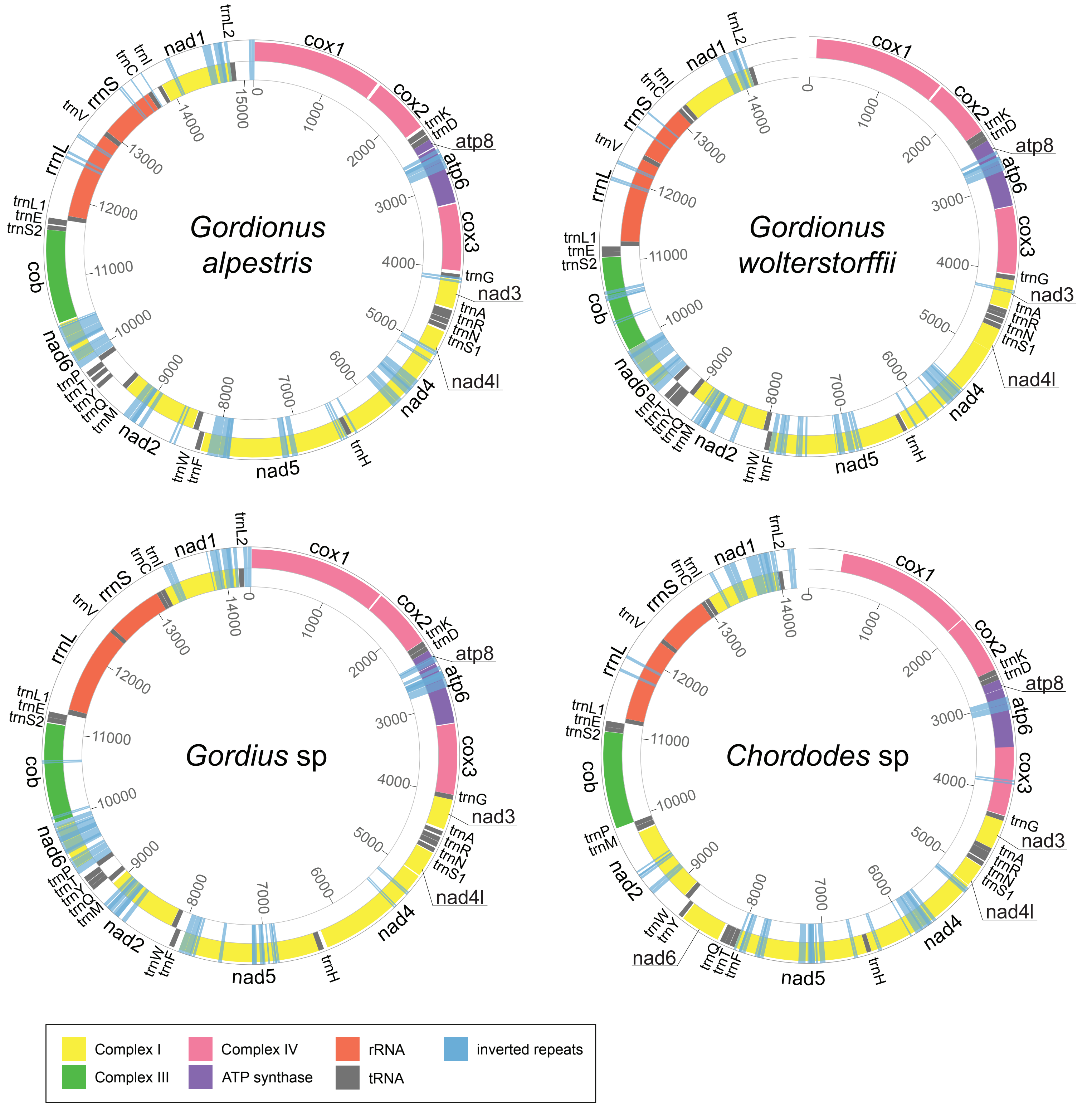

Figure S1. Assemblies and annotations of the mitochondrial genomes of the four sequenced species of Nematomorpha. NCBI GenBank accession numbers: *Gordionus alpestris*, MG257765; *Gordionus wolterstorffii*, MG257766; *Gordius* sp., MG257767; *Chordodes* sp., MG257764.

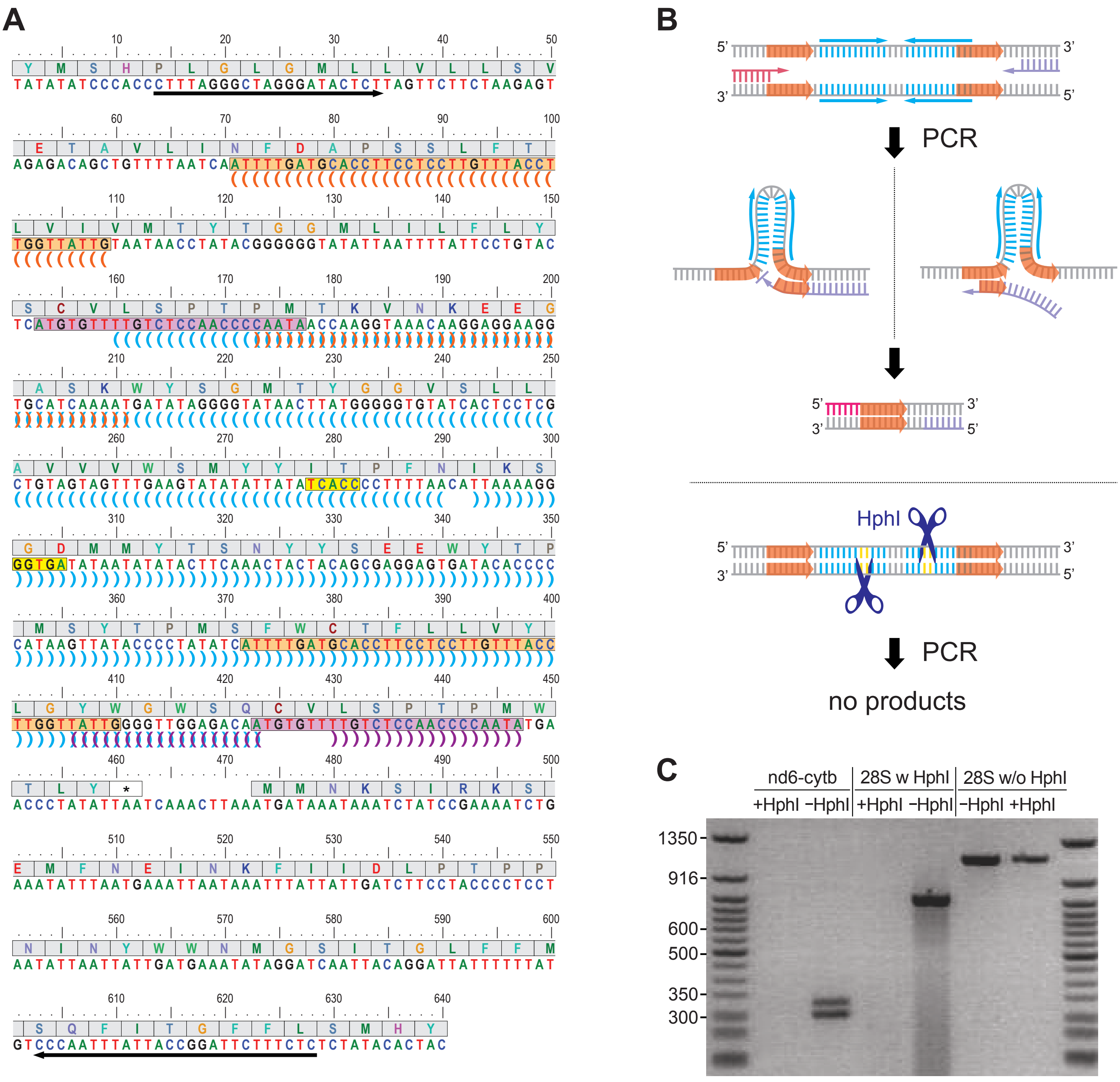

Figure S2. Validation of the hairpin sequence using HphI restriction. (**A**) Fragment of the *G. alpestris* mtDNA assembly featuring partial sequences of nad6 and cob genes and a system of three overlapping inverted repeats (indicated with parentheses). The longest hairpin (blue) is flanked on each side by shorter hairpins (orange and purple) that overlap the main hairpin with one of their arms, thus forming two direct repeats in the sequence – indicated with orange and purple highlighter in the nucleotide sequence. The primer sequences used for PCR are indicated with black arrows, and the HphI restriction site is highlighted in yellow. (**B**) PCR with the designed primers does not recover the expected 615 bp sequence predicted by the assembly, producing instead shorter products corresponding to the two possible skips of long palindrome by means of flanking direct repeats. To confirm that the shorter PCR products are in fact artefactual, we used the HphI restriction enzyme to cut at a site (TCACC, GGTGA) present in our assembly but not in the actual amplified sequences; if the amplified sequences occur naturally and our assembly is incorrect, this procedure should not affect the outcome of PCR. (**C**) The result of PCR confirms that HphI enzyme treatment (11 hours, 37°C) prevents product amplification. The two shorter products without the main hairpin (345 and 314 bp) could not be amplified after the restrictase treatment. For control we used the *G. alpestris* 28S rRNA gene. Fragments of the gene (803 and 1083 bp) with or without the HphI restriction site were amplified with the following sets of primers: with the restriction site - 28ddF GTCTTGAAACACGGACCAAGGAGTCT and 28EEAR CGCGACTCTCCTACTCGTTCGA; without the restriction site - 28eeF ATCCGCTAAGGAGTGTGTAACAACTCACC and 28ggR GATGACGAGGCATTTGGCTACC.

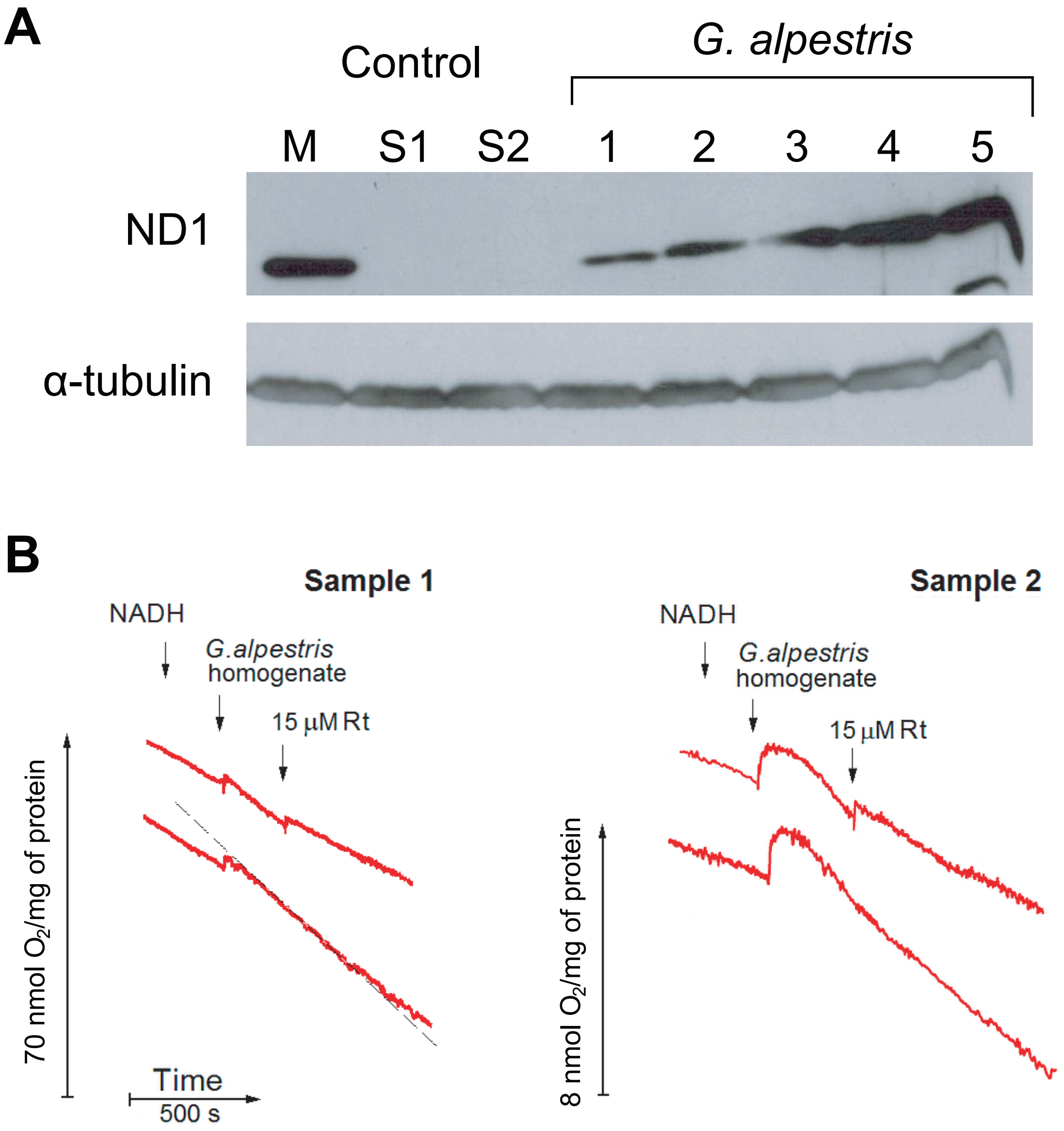

Figure S3. Immunostaining of *G. alpestris* nad1 and respiratory activity measurements. (**A**) Western blot of *G. alpestris* homogenates (1-5) with anti-ND1 antibodies; M - rat liver mitochondria (positive control) obtained by differential centrifugation; S1 and S2 are supernatants from different steps of mitochondria purification procedure at 9000g and correspond to cytosol (negative control). (**B**) Oxygen consumption in the *G. alpestris* tissue homogenates. The two panels represent measurements for specimens extracted directly from the host (Sample 1) or captured in the water (Sample 2). The incubation mixture for each variant contained alamethicin 50 µg/mL and 200 µM NADH. Oxygen consumption in the incubation mixture was triggered by the addition of *G. alpestris* tissue homogenate to achieve the final concentration of 1.3 (sample 1) or 3 (sample 2) mg of protein per mL. Rt: the inhibitor of complex I, rotenone, which was added to the incubation mixtures as shown in the upper curves of both panels after the respiration had begun; the lower curves are the records without rotenone addition; arrows point the moments of the above listed additions.

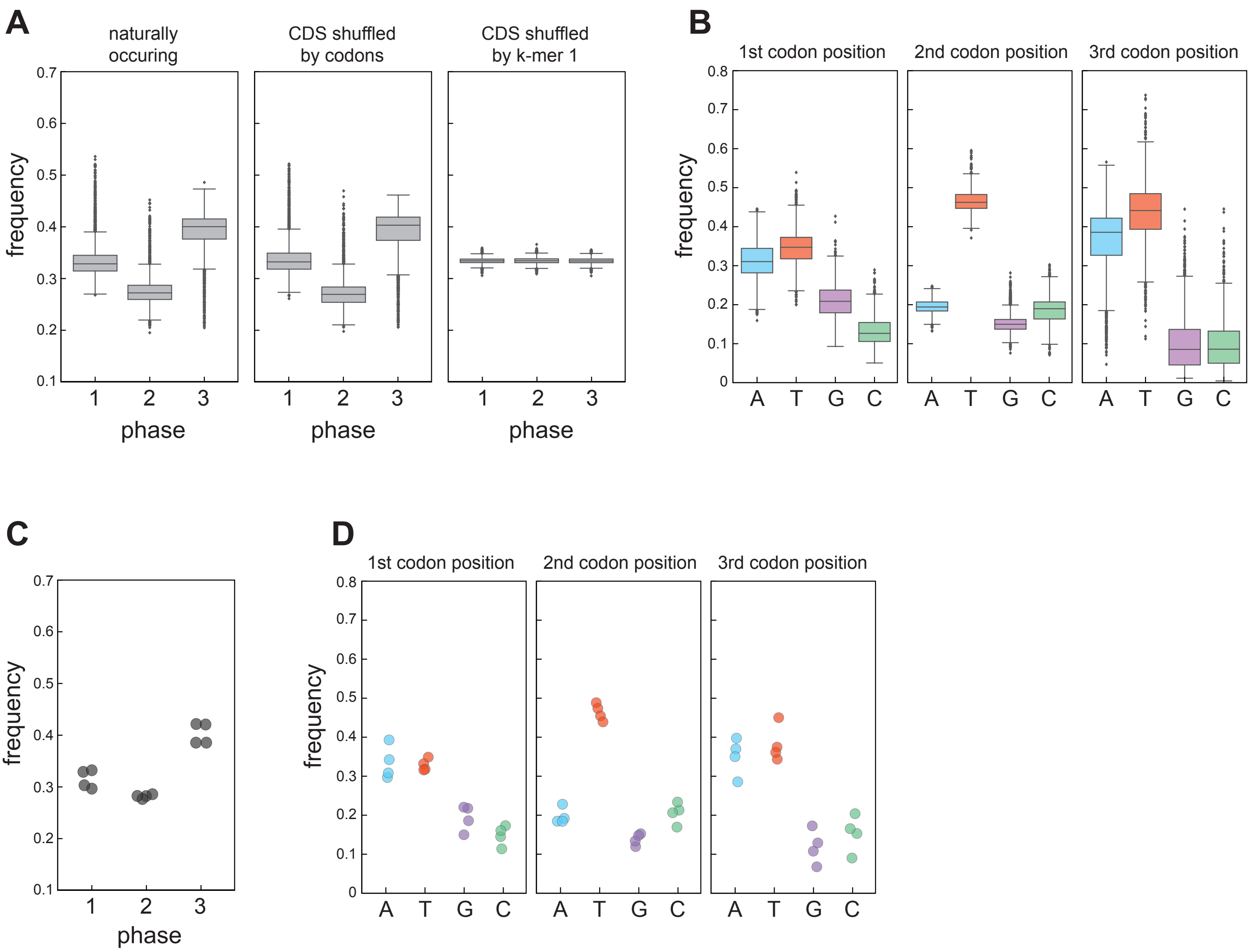

Figure S4. Distributions of inverted repeat phase proportions in the coding sequences of mitochondrial genomes of invertebrate animals. (**A**) Frequencies of inverted repeats >5 bp of each phase in the mitochondrial genomes of invertebrates (3,103 mt genomes), each data point – summarized repeat counts for the genome; the counts were performed for the actual coding sequences (CDS) (leftmost plot) and for CDS with randomized sequences: the sequences were randomized by shuffling codons (central plot), which preserves the original codon frequencies, and by shuffling nucleotides (rightmost plot), which preserves only the nucleotide composition of sequences. The plots demonstrate that the codon frequencies alone, but not nucleotide composition, are sufficient to produce the repeat phase proportions observed in the actual CDS. (**B**) Nucleotide composition by codon position in the CDS of invertebrate mitochondrial genomes. (**C**) The observed frequencies of inverted repeats >5 bp of each phase in the mitochondrial genomes of Nematomorpha (4 data points); (**D**) Nucleotide composition in the CDS of nematomorph mitochondrial genomes. Both the repeat phase proportions for repeats >5 bp and the nucleotide compositions in nematomorphs show good agreement with data from other invertebrates.

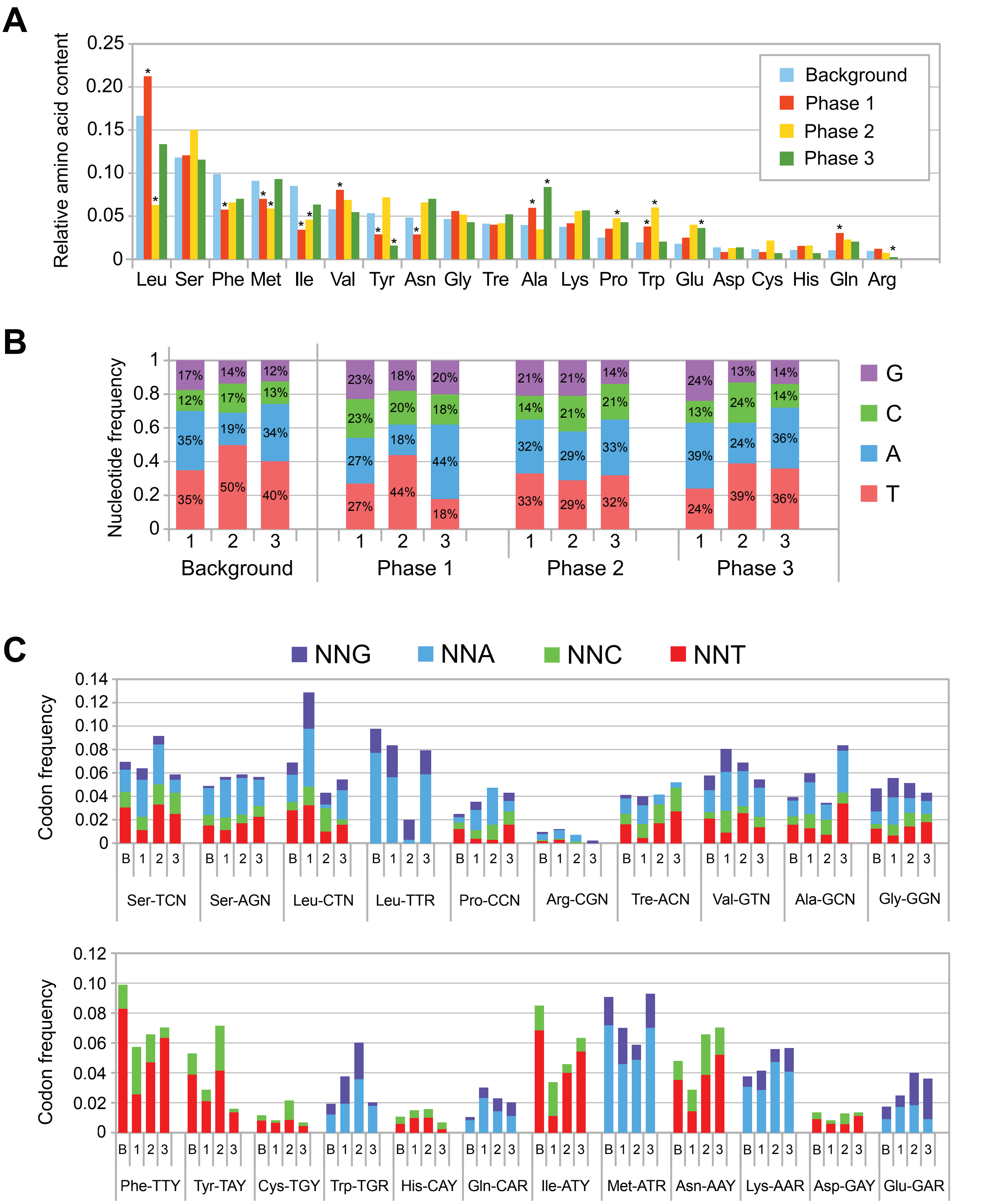

Figure S5. Nucleotide and amino acid content of inverted repeat regions in the mitochondrial genes of Nematomorpha. (**A**) Amino acid composition of translated genes partitioned into repeat-containing (phases 1-3) and non-repeat regions (background); asterisk denotes amino acid content with significantly skewed composition in the repeat region according to the Composition Profiler ($P < 0.05$, Bonferroni adjustment, comparison to background sequence). (**B**) Nucleotide content by codon position for the non-repeat gene regions (background) and repeat-containing regions (phases 1-3). (**C**) Histograms of codon usage in the non-repeat gene regions - "B", and repeat-containing regions (phases 1-3).

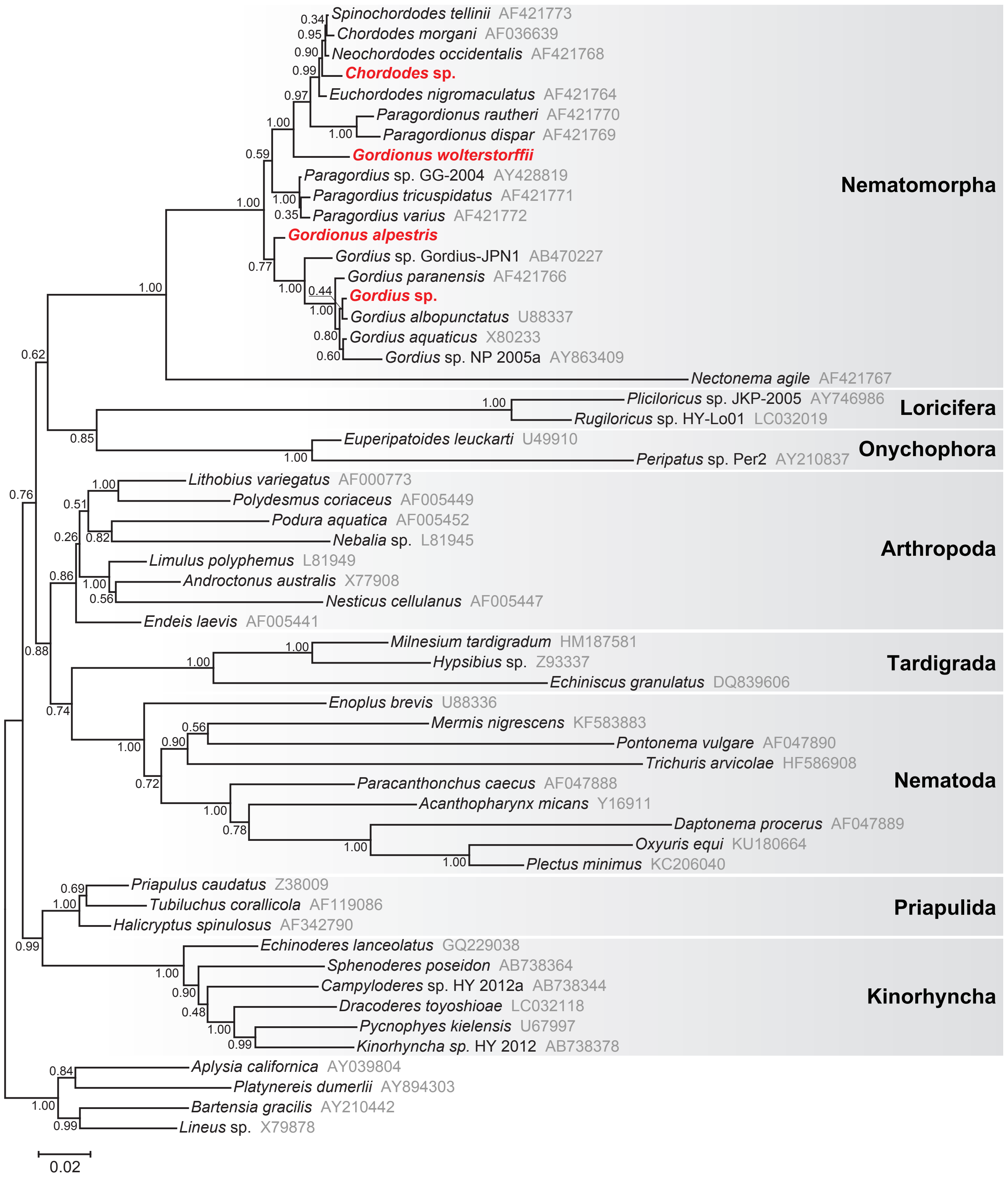

Figure S6. Bayesian inference with 18S rRNA genes. The tree features major ecdysozoan taxa (labeled) and is rooted with sequences of lophotrochozoans. The sequence names are provided with NCBI database accessions; the four nematomorph species analyzed in this study are given in red. Node support values are posterior probabilities summarized by MrBayes from 4 independent runs of 10M generations with a 50% burn-in.

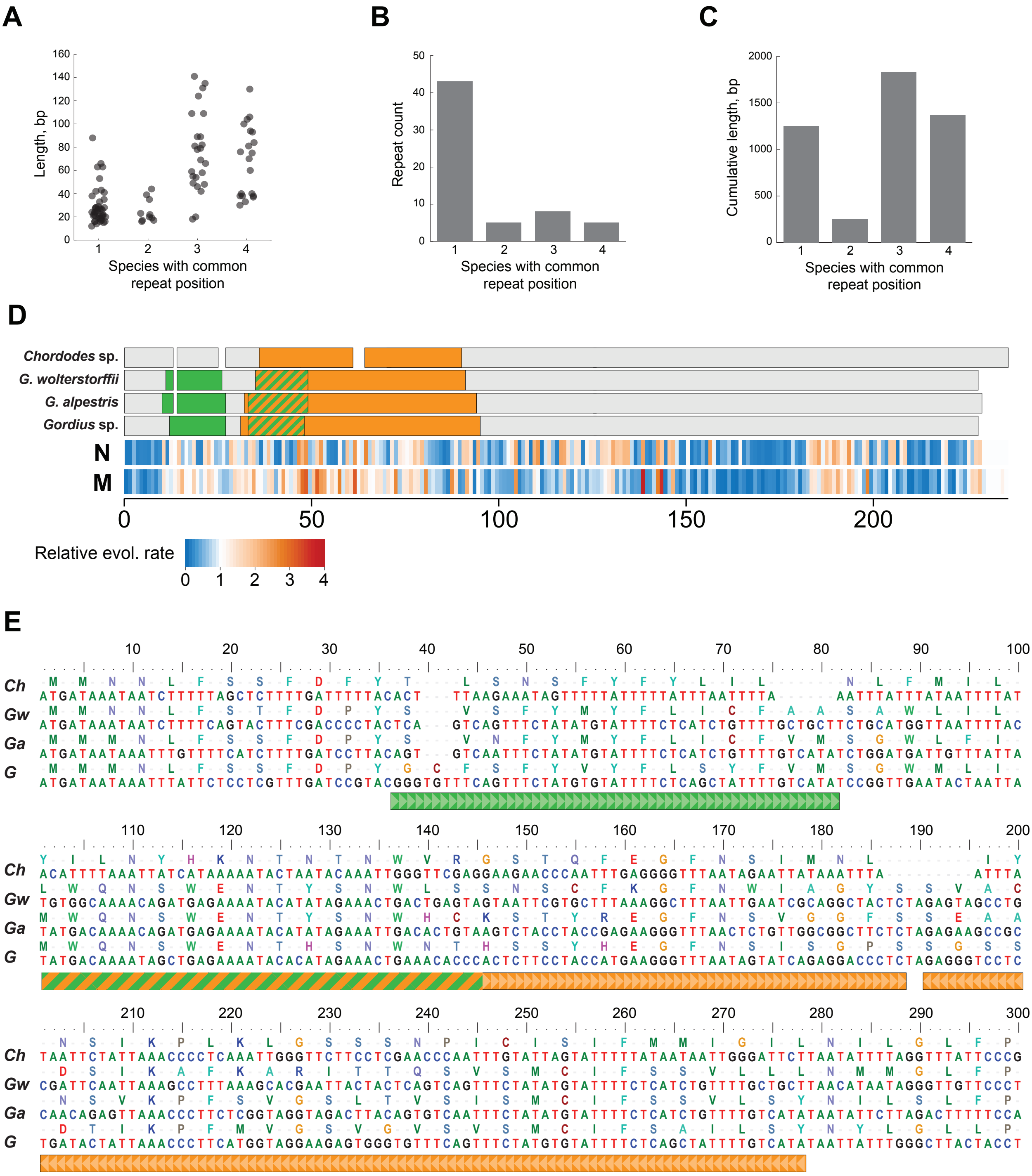

Figure S7. Characteristics of shared inverted repeats in the mitochondrial genomes of Nematomorpha. (**A**) Categorical plot of inverted repeat lengths by the number species that have the repeat; the inverted repeat was considered as shared by two or more species if its midpoint position (between the arms of the repeat) in the alignment coincided in these species; the repeats for each species were considered here individually, i.e. a repeat shared by all four species of nematomorphs is featured four times in the plot. (**B**) Non-redundant counts of repeats of each type – the shared repeat counts normalized by the number of shared species. (**C**) Summarized lengths of repeats of each type; the repeats were once again summarized for each species individually due to interspecies variation in shared repeat lengths. (**D**) Schematic representation of an alignment of atp6 sequences from nematomorph genomes; the inverted repeat regions are highlighted in color (phase 1 repeat – green; phase 2 repeat – orange); the overlapping repeat region is indicated with a striped pattern. The relative site rate estimates for the alignment positions were evaluated for two variants of the alignment: a variant using only the four nematomorph sequences – “N”, and a variant using a set of ~40 metazoan sequences – “M”; the relative rate estimates were obtained individually for each dataset and are not directly comparable between datasets. However, the conservation profiles demonstrate that the presence of a hairpin in the sequence puts additional constraints on the variability in the region, but does not completely restrict its mutability. (**E**) A detailed portion of the atp6 gene alignment (positions 1-100 in D) with the corresponding nucleotide and amino acid sequences and the approximate layout of hairpin regions.

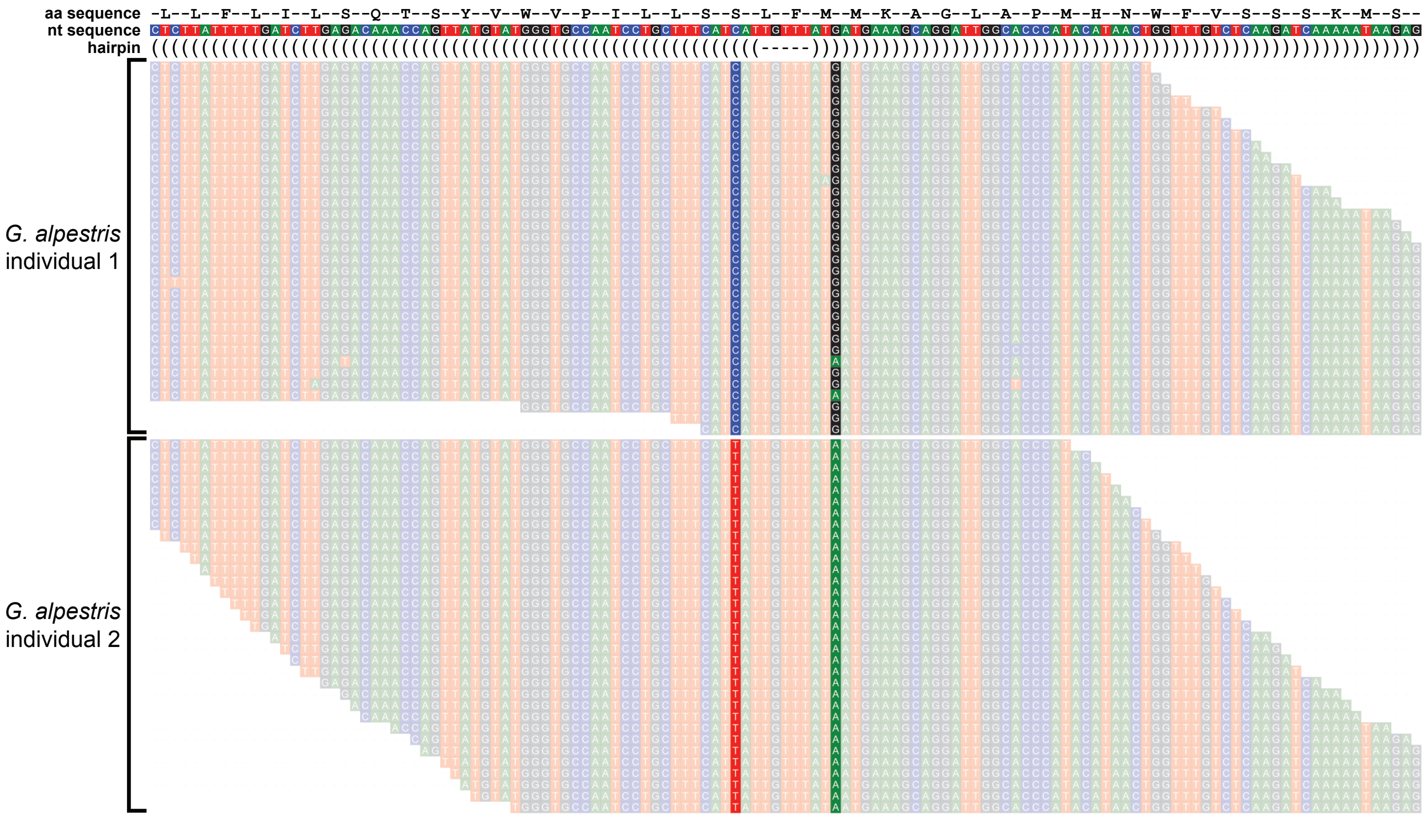

Figure S8. Sequence variation in the complementary arms of the inverted repeat. Reads from the libraries of two individuals of *G. alpestris* (one sequenced with MiSeq and the other with HiSeq) were mapped to the reference sequence (top) with bowtie2; the figure features an alignment for the nad2 hairpin region – the reference is based on the sequence of the first individual; the corresponding amino acid sequence and the inverted repeat structure in the bracket notation are given above the alignments. The complementary substitutions are highlighted in the alignments.

Table S1. Nucleotide composition characteristics of hairworm mitochondrial genomes.

Species	GC%	A%	T%	G%	C%	AT-skew	GC-skew
Gordionus alpestris	29	33	38	12	17	-0.07	-0.17
Gordionus wolterstorffii	35	30	35	17	18	-0.08	-0.03
Gordius sp.	34	31	35	15	19	-0.06	-0.12
Chordodes sp.	22	37	41	10	12	-0.05	-0.09

Table S2. GC% content of hairworm mitochondrial genomes.

Species	Whole genome	PCG	tRNA	rRNA	Non-coding regions
Gordionus alpestris	29	31	24	25	26
Gordionus wolterstorffii	35	37	27	28	33
Gordius sp.	34	35	33	31	33
Chordodes sp.	22	23	19	19	20

Table S3. Differential expression analysis of RNA-Seq data for the *G. alpestris* specimens extracted from the host (H) or captured free from the host (F); genes containing inverted repeats are labeled with brackets.

	RNA-Seq read alignment count						EdgeR exact test (F-H)			
Gene	H1	H2	H3	F1	F2	F3	logFC	logCPM	p-value	FDR
cox1	1825	2014	12209	19905	25753	18630	-0.463	17.890	0.210	0.588
cox2	1090	1235	7552	15699	20616	15666	-0.057	17.359	0.878	0.983
atp8	2	9	501	202	308	1135	0.477	12.177	0.561	0.983
atp6 [ ]	142	128	2700	4948	6254	7669	0.974	15.455	0.027	0.350
cox3	1510	1204	7740	24190	19431	13709	-0.036	17.539	0.923	0.983
nad3	9	11	423	236	696	1955	1.260	12.668	0.136	0.588
nad4l	5	1	283	120	419	853	1.039	11.764	0.226	0.588
nad4 [ ]	281	191	2675	4089	3410	5259	-0.010	15.312	0.983	0.983
nad5 [ ]	195	191	4897	5680	7481	11135	0.628	15.926	0.126	0.588
nad2 [ ]	538	487	5779	5088	11649	8548	-0.188	16.347	0.628	0.983
nad6 [ ]	73	16	906	565	712	1254	-0.332	13.129	0.690	0.983
cob	561	801	8475	9986	12214	16210	-0.041	16.892	0.915	0.983
nad1 [ ]	245	146	1186	3474	3920	1473	0.052	14.794	0.919	0.983

Table S4. Perfect inverted repeats over the length of 20 bp in the coding sequences of mitochondrial genomes. Only the maximal repeat for each CDS is featured in the table.

Repeat Length	Repeat Distance	Phase	AT content %	Dust Masker %	CDS	Genome Accession	Lineage	Species
146	1	3	76	0	orf1097	NC_034798.1	Heterolobosea; Pharyngomonas	*Pharyngomonas kirbyi*
87	2	1	57	0	orf_290	LT671462.1	Metazoa; Myxosporea	*Kudoa iwatai*
61	5	3	57	0	ND6	NC_026985.1	Metazoa; Gastrotricha	*Lepidodermella squamata*
59	1	1	53	0	ND5	NC_026985.1	Metazoa; Gastrotricha	*Lepidodermella squamata*
46	11	1	65	0	ND1	NC_026985.1	Metazoa; Gastrotricha	*Lepidodermella squamata*
46	8	3	70	0	ND2	NC_026985.1	Metazoa; Gastrotricha	*Lepidodermella squamata*
28	0	3	50	0	COX2	NC_026985.1	Metazoa; Gastrotricha	*Lepidodermella squamata*
58	2	3	72	0	orf819	NC_013986.1	Amoebozoa; Tubulinea	*Hartmannella vermiformis*
52	0	1	69	0	orf901	NC_013986.1	Amoebozoa; Tubulinea	*Hartmannella vermiformis*
32	10	3	78	0	orf184	NC_013986.1	Amoebozoa; Tubulinea	*Hartmannella vermiformis*
28	0	1	79	25	orf552	NC_013986.1	Amoebozoa; Tubulinea	*Hartmannella vermiformis*
58	70	1	67	0	ND2	NC_006160.1	Metazoa; Arthropoda	*Aleurochiton aceris*
46	158	3	26	0	VAR1	CP006534.1	Fungi; Ascomycota	*Saccharomyces cerevisiae*
21	1061	3	95	0	AI5 beta	CP004115.1	Fungi; Ascomycota	*Saccharomyces cerevisiae*
21	1028	3	95	0	AI5 beta	CP006508.1	Fungi; Ascomycota	*Saccharomyces cerevisiae*
21	1061	3	95	0	AI5 beta	CP006520.1	Fungi; Ascomycota	*Saccharomyces cerevisiae*
21	1022	3	95	0	AI5 beta	CP006529.1	Fungi; Ascomycota	*Saccharomyces cerevisiae*
21	1061	3	95	0	AI5 beta	LBMA01000018.1	Fungi; Ascomycota	*Saccharomyces cerevisiae*
41	32	3	80	17	ND2	NC_028200.1	Metazoa; Platyhelminthes	*Hoploplana elisabelloi*
40	45	1	80	0	ND5	NC_033868.1	Metazoa; Nematoda	*Paralongidorus litoralis*
22	0	1	64	0	ND6	NC_033868.1	Metazoa; Nematoda	*Paralongidorus litoralis*
37	64	1	89	19	ND5	NC_008640.1	Metazoa; Nematoda	*Romanomermis culicivorax*
30	1	3	90	0	ND2	NC_008640.1	Metazoa; Nematoda	*Romanomermis culicivorax*
21	4	3	81	0	ND4	NC_008640.1	Metazoa; Nematoda	*Romanomermis culicivorax*
36	845	1	64	0	rps7	NC_036614.1	Chlorophyta; Pyramimonadales	*Cymbomonas tetramitiformis*
27	497	2	81	0	rps2	NC_036614.1	Chlorophyta; Pyramimonadales	*Cymbomonas tetramitiformis*
35	12	1	49	0	rps2	NC_027722.1	Chlorophyta; Trebouxiophyceae	*Botryococcus braunii*
31	290	3	39	0	rps3	NC_027722.1	Chlorophyta; Trebouxiophyceae	*Botryococcus braunii*
29	1053	3	38	0	orf1930	NC_027722.1	Chlorophyta; Trebouxiophyceae	*Botryococcus braunii*
25	12	1	48	0	ND5	NC_027722.1	Chlorophyta; Trebouxiophyceae	*Botryococcus braunii*
32	1	3	66	0	ND4	NC_033870.1	Metazoa; Nematoda	*Xiphinema pachtaicum*
21	0	2	62	0	ND5	NC_033870.1	Metazoa; Nematoda	*Xiphinema pachtaicum*
32	0	1	66	0	ND1	NC_020323.1	Metazoa; Arthropoda	*Liphistius erawan*
32	0	1	69	0	ND2	NC_037488.1	Metazoa; Arthropoda	*Diaphanosoma dubium*
30	33	1	87	0	ND5	NC_008046.1	Metazoa; Nematoda	*Thaumamermis cosgrovei*
30	1	2	90	23	ND5	NC_014505.1	Metazoa; Arthropoda	*Tanystylum orbiculare*
30	1004	1	87	0	rps4	NC_026009.1	Chlorophyta; Trebouxiophyceae	*Auxenochlorella protothecoides*
29	1	3	66	0	COX3	NC_039402.1	Metazoa; Arthropoda	*Parhyale hawaiensis*
28	90	1	79	0	COX3	NC_008828.1	Metazoa; Nematoda	*Hexamermis agrotis*
28	29	3	93	0	CYTB	NC_016017.1	Metazoa; Arthropoda	*Stenopirates sp.*
28	0	3	68	0	ND4	NC_025511.1	Metazoa; Mollusca	*Camaena cicatricosa*
28	633	3	75	0	rps4	NC_025413.1	Chlorophyta; Trebouxiophyceae	*Chlorella variabilis*
27	51	3	67	0	CYTB	NC_021141.1	Metazoa; Platyhelminthes	*Hydatigera parva*
27	6	3	93	0	ND5	NC_008692.1	Metazoa; Nematoda	*Romanomermis nielseni*
27	0	3	63	0	CYTB	NC_002176.1	Metazoa; Mollusca	*Pupa strigosa*
27	249	2	70	0	orf114c	NC_002511.2	Streptophyta; Tracheophyta	*Beta vulgaris*
27	249	2	70	0	orf114c	NC_015099.1	Streptophyta; Tracheophyta	*Beta vulgaris*
27	249	2	70	0	orf114c	NC_015994.1	Streptophyta; Tracheophyta	*Beta macrocarpa*
22	29	1	50	0	orf317	NC_002511.2	Streptophyta; Tracheophyta	*Beta vulgaris*
22	29	1	50	0	orf317	NC_015099.1	Streptophyta; Tracheophyta	*Beta vulgaris*
22	29	1	50	0	orf317	NC_015994.1	Streptophyta; Tracheophyta	*Beta macrocarpa*
26	51	3	73	0	CYTB	NC_014768.1	Metazoa; Platyhelminthes	*Taenia taeniaeformis*
26	51	3	69	0	CYTB	NC_021142.1	Metazoa; Platyhelminthes	*Hydatigera krepkogorski*
26	51	3	65	0	CYTB	NC_037071.1	Metazoa; Platyhelminthes	*Hydatigera kamiyai*
26	23	3	77	0	ND6	NC_006078.1	Metazoa; Arthropoda	*Ixodes uriae*
26	0	2	58	0	ND3	NC_034226.1	Metazoa; Mollusca	*Cerion uva*
25	2049	1	52	0	polymerase	NC_035155.1	Fungi; Ascomycota	*Sclerotinia sclerotiorum*
25	25	3	76	0	orf163	NC_020354.1	Fungi; Basidiomycota	*Microbotryum cf. violaceum*
24	3	2	92	96	ND5	NC_020354.1	Fungi; Basidiomycota	*Microbotryum cf. violaceum*
25	11	3	88	0	ATP6	NC_017759.1	Metazoa; Arthropoda	*Aponomma fimbriatum*
21	11	1	76	0	ND3	NC_017759.1	Metazoa; Arthropoda	*Aponomma fimbriatum*
25	11	3	84	28	ATP6	NC_026552.1	Metazoa; Arthropoda	*Dermacentor silvarum*
25	29	3	68	0	orf101	NC_005926.1	Chlorophyta; Ulvophyceae	*Pseudendoclonium akinetum*
23	536	3	35	0	orf233	NC_005926.1	Chlorophyta; Ulvophyceae	*Pseudendoclonium akinetum*
21	29	3	67	0	orf115a	NC_005926.1	Chlorophyta; Ulvophyceae	*Pseudendoclonium akinetum*
21	0	3	43	0	orf63	NC_005926.1	Chlorophyta; Ulvophyceae	*Pseudendoclonium akinetum*
25	0	1	88	72	ccmFC	NC_024521.1	Streptophyta; Bryophyta	*Sphagnum palustre*
24	0	2	96	0	ND2	NC_017745.1	Metazoa; Arthropoda	*Amblyomma sphenodonti*
22	11	3	86	0	ATP6	NC_017745.1	Metazoa; Arthropoda	*Amblyomma sphenodonti*
24	8	1	83	0	ND5	NC_017756.1	Metazoa; Arthropoda	*Bothriocroton concolor*
21	15	3	90	0	ATP6	NC_017756.1	Metazoa; Arthropoda	*Bothriocroton concolor*
24	11	3	83	29	ATP6	NC_028528.1	Metazoa; Arthropoda	*Dermacentor nuttalli*
24	238	1	100	0	ND4	NC_035883.1	Metazoa; Arthropoda	*Apis mellifera sahariensis*

24	6	3	88	0	ND2	NC_034842.1	Metazoa; Arthropoda	*Neostylopyga rhombifolia*
24	15	3	83	0	ND6	NC_020775.1	Metazoa; Arthropoda	*Lithidiopsis carinatus*
24	24	3	79	0	ND3	NC_012644.1	Metazoa; Arthropoda	*Davidius lunatus*
23	4	3	83	0	orf265	NC_026312.1	Heterolobosea; Schizopyrenida	*Stachyamoeba lipophora*
23	197	3	100	0	ND6	NC_013147.1	Fungi; Ascomycota	*Dekkera bruxellensis*
23	246	3	96	0	rps3	NC_027459.1	Fungi; Ascomycota	*Torulaspora globosa*
23	13	3	83	0	ATP6	NC_020335.1	Metazoa; Arthropoda	*Haemaphysalis parva*
23	31	2	35	30	ND4	NC_000844.1	Metazoa; Arthropoda	*Daphnia pulex*
23	16	3	96	0	ATP6	NC_026218.1	Metazoa; Arthropoda	*Colletes gigas*
23	30	1	100	100	ND4	NC_018545.1	Metazoa; Arthropoda	*Mengenilla moldrzyki*
21	31	1	100	100	ND4	NC_018545.1	Metazoa; Arthropoda	*Mengenilla moldrzyki*
23	102	1	48	0	COX3	NC_029155.1	Metazoa; Arthropoda	*Aleurocanthus spiniferus*
23	197	3	61	0	ND6	NC_029155.1	Metazoa; Arthropoda	*Aleurocanthus spiniferus*
23	118	2	78	0	ND5	NC_015999.1	Metazoa; Arthropoda	*Ibidoecus bisignatus*
21	42	3	71	0	ND2	NC_015999.1	Metazoa; Arthropoda	*Ibidoecus bisignatus*
23	77	3	78	0	ND2	NC_030267.1	Metazoa; Arthropoda	*Creobroter gemmatus*
22	8	3	64	0	COX2	NC_030267.1	Metazoa; Arthropoda	*Creobroter gemmatus*
23	8	2	78	0	orf37	NC_007685.1	Stramenopiles; Phaeophyceae	*Dictyota dichotoma*
22	15	3	82	0	ATP6	NC_020333.1	Metazoa; Arthropoda	*Amblyomma cajennense*
22	15	3	82	0	ATP6	NC_032369.1	Metazoa; Arthropoda	*Amblyomma sculptum*
21	77	2	100	33	ND6	NC_020333.1	Metazoa; Arthropoda	*Amblyomma cajennense*
21	77	2	100	33	ND6	NC_032369.1	Metazoa; Arthropoda	*Amblyomma sculptum*
22	1	1	68	0	ND5	NC_014687.1	Metazoa; Arthropoda	*Caprella scaura*
22	719	3	95	0	ND5	NC_034676.1	Metazoa; Arthropoda	*Burara striata*
22	59	2	91	36	ATP8	NC_026198.1	Metazoa; Arthropoda	*Melipona scutellaris*
22	77	3	82	0	ND2	NC_037234.1	Metazoa; Arthropoda	*Creobroter jiangxiensis*
22	8	3	59	0	COX2	NC_037204.1	Metazoa; Arthropoda	*Sphodromantis lineola*
22	8	3	64	0	COX2	NC_030266.1	Metazoa; Arthropoda	*Tenodera sinensis*
22	8	3	64	0	COX2	NC_037697.1	Metazoa; Arthropoda	*Paratoxodera polyacantha*
22	8	3	64	0	COX2	NC_037381.1	Metazoa; Arthropoda	*Toxodera hauseri*
22	414	1	41	0	ND2	NC_026104.1	Metazoa; Arthropoda	*Acroneuria hainana*
21	21	3	100	48	Ymf76	NC_015981.1	Alveolata; Ciliophora	*Ichthyophthirius multifiliis*
21	0	2	100	0	ymf77	NC_008339.1	Alveolata; Ciliophora	*Tetrahymena pigmentosa*
21	243	3	100	0	yejR	NC_014262.1	Alveolata; Ciliophora	*Paramecium caudatum*
21	26	3	95	0	rps3	NC_006862.1	Amoebozoa; Mycetozoa	*Polysphondylium pallidum*
21	1336	3	100	0	Mp36-like	NC_010653.1	Amoebozoa; Mycetozoa	*Dictyostelium fasciculatum*
21	42	1	86	76	GIY-YIG	NC_025200.1	Fungi; Ascomycota	*Sclerotinia borealis*
21	192	3	81	0	cox1-I3	NC_022159.1	Fungi; Ascomycota	*Barnettozyma californica*
21	989	3	95	0	AI5 beta	NC_001224.1	Fungi; Ascomycota	*Saccharomyces cerevisiae*
21	0	1	95	0	orf314	NC_037774.1	Fungi; Basidiomycota	*Russula abietina*
21	15	3	81	0	ATP6	NC_005292.1	Metazoa; Arthropoda	*Haemaphysalis flava*
21	15	3	76	0	ATP6	NC_020334.1	Metazoa; Arthropoda	*Haemaphysalis formosensis*
21	15	3	81	0	ATP6	NC_037246.1	Metazoa; Arthropoda	*Haemaphysalis japonica*
21	15	3	86	0	ATP6	NC_037493.1	Metazoa; Arthropoda	*Haemaphysalis longicornis*
21	11	3	81	33	ATP6	NC_023349.1	Metazoa; Arthropoda	*Dermacentor nitens*
21	15	3	86	0	ATP6	NC_002074.1	Metazoa; Arthropoda	*Rhipicephalus sanguineus*
21	439	3	100	0	ND2	NC_021410.1	Metazoa; Arthropoda	*Ctenoplusia agnata*
21	439	3	100	0	ND2	NC_025760.1	Metazoa; Arthropoda	*Ctenoplusia limbirena*
21	26	2	100	0	ATP8	NC_027443.1	Metazoa; Arthropoda	*Spoladea recurvalis*
21	437	1	90	0	ND4	NC_029497.1	Metazoa; Arthropoda	*Acraea egina*
21	510	3	76	0	ND5	NC_013254.1	Metazoa; Arthropoda	*Mordella atrata*
21	165	3	95	0	ND2	NC_019595.1	Metazoa; Arthropoda	*Gorpis annulatus*
21	36	3	100	0	ND2	NC_021975.1	Metazoa; Arthropoda	*Lygus lineolaris*
21	3	1	95	33	ND2	NC_035510.1	Metazoa; Arthropoda	*Anaphothrips obscurus*
21	8	3	62	0	COX2	NC_034284.1	Metazoa; Arthropoda	*Rhombodera valida*
21	8	3	52	0	COX2	NC_037235.1	Metazoa; Arthropoda	*Sibylla pretiosa*
21	0	3	71	0	ND1	NC_010432.1	Metazoa; Entoprocta	*Loxosomella aloxiata*
21	10	1	100	33	ATP4	NC_032002.1	Rhodophyta; Florideophyceae	*Choreocolax polysiphoniae*
21	178	3	90	0	CP76_p10	NC_023784.1	Rhodophyta; Florideophyceae	*Gracilaria salicornia*
21	163	1	81	67	orf151	NC_029886.1	Collodictyonidae	*Diphylleia rotans*
21	0	2	33	0	orf125_1	NC_013765.1	Streptophyta; Anthocerotophyta	*Phaeoceros laevis*
21	0	3	90	38	ccmFC	NC_024519.1	Streptophyta; Bryophyta	*Bartramia pomiformis*
21	0	3	86	38	ccmFC	NC_036945.1	Streptophyta; Bryophyta	*Mielichhoferia elongata*
21	0	3	90	38	ccmFC	NC_026121.1	Streptophyta; Bryophyta	*Orthotrichum speciosum*
21	0	3	90	38	ccmFC	NC_031391.1	Streptophyta; Bryophyta	*Nyholmiella gymnostoma*
21	0	3	90	38	ccmFC	NC_031767.1	Streptophyta; Bryophyta	*Nyholmiella obtusifolia*
21	0	3	90	38	ccmFC	NC_024522.1	Streptophyta; Bryophyta	*Orthotrichum stellatum*
21	0	3	90	38	ccmFC	NC_029355.1	Streptophyta; Bryophyta	*Orthotrichum macrocephalum*
21	0	3	90	38	ccmFC	NC_029356.1	Streptophyta; Bryophyta	*Orthotrichum diaphanum*
21	0	3	90	38	ccmFC	NC_031389.1	Streptophyta; Bryophyta	*Orthotrichum bicolor*
21	0	3	90	38	ccmFC	NC_031390.1	Streptophyta; Bryophyta	*Orthotrichum callistomum*
21	0	3	90	38	ccmFC	NC_031392.1	Streptophyta; Bryophyta	*Stoneobryum bunyaense*
21	0	3	90	38	ccmFC	NC_034907.1	Streptophyta; Bryophyta	*Stoneobryum mirum*
21	0	3	90	38	ccmFC	NC_024517.1	Streptophyta; Bryophyta	*Ulota hutchinsiae*
21	0	3	90	38	ccmFC	NC_031393.1	Streptophyta; Bryophyta	*Ulota crispa*
21	0	3	90	38	ccmFC	NC_031394.1	Streptophyta; Bryophyta	*Ulota phyllantha*
21	0	3	90	38	ccmFC	NC_031846.1	Streptophyta; Bryophyta	*Zygodon viridissimus*
21	0	3	90	38	ccmFC	NC_028191.1	Streptophyta; Bryophyta	*Tetraplodon fuegianus*
21	0	3	90	38	ccmFC	NC_027974.1	Streptophyta; Bryophyta	*Sanionia uncinata*
21	0	3	86	38	ccmFC	NC_016121.1	Streptophyta; Bryophyta	*Anomodon rugelii*

21	0	3	90	38	ccmFC	NC_031212.1	Streptophyta; Bryophyta	*Brachythecium rivulare*
21	0	3	90	38	ccmFC	NC_024515.1	Streptophyta; Bryophyta	*Climacium americanum*
21	0	3	90	38	ccmFC	NC_024516.1	Streptophyta; Bryophyta	*Hypnum imponens*
21	0	3	90	38	ccmFC	NC_024514.1	Streptophyta; Bryophyta	*Ptychomnion cygnisetum*
21	0	3	95	38	ccmFC	NC_024518.1	Streptophyta; Bryophyta	*Buxbaumia aphylla*
21	0	3	90	38	ccmFC	NC_026974.1	Streptophyta; Bryophyta	*Bucklandiella orthotrichacea*
21	0	3	90	38	ccmFC	NC_026784.1	Streptophyta; Bryophyta	*Codriophorus aciculare*
21	0	3	90	38	ccmFC	NC_025931.1	Streptophyta; Bryophyta	*Codriophorus laevigatus*
21	0	3	90	38	ccmFC	NC_026891.1	Streptophyta; Bryophyta	*Codriophorus varius*
21	0	3	90	38	ccmFC	NC_026540.1	Streptophyta; Bryophyta	*Racomitrium ericoides*
21	0	3	90	38	ccmFC	NC_026890.1	Streptophyta; Bryophyta	*Racomitrium elongatum*
21	0	3	90	38	ccmFC	NC_026975.1	Streptophyta; Bryophyta	*Racomitrium emersum*
21	0	3	90	38	ccmFC	NC_029452.1	Streptophyta; Bryophyta	*Racomitrium lanuginosum*
21	0	3	90	38	ccmFC	NC_028040.1	Streptophyta; Bryophyta	*Oxystegus tenuirostris*
21	0	3	90	38	ccmFC	NC_027515.1	Streptophyta; Bryophyta	*Syntrichia filaris*
21	0	3	90	38	ccmFC	NC_024523.1	Streptophyta; Bryophyta	*Funaria hygrometrica*
21	0	3	90	38	ccmFC	NC_007945.1	Streptophyta; Bryophyta	*Physcomitrella patens*
21	0	2	38	0	PlpuMp08	NC_013444.1	Streptophyta; Marchantiophyta	*Pleurozia purpurea*
21	11	3	52	0	PlpuMp42	NC_013444.1	Streptophyta; Marchantiophyta	*Pleurozia purpurea*
